# Lysine methyltransferase SMYD2 promotes triple negative breast cancer progression

**DOI:** 10.1038/s41419-018-0347-x

**Published:** 2018-02-27

**Authors:** Linda Xiaoyan Li, Julie Xia Zhou, James P. Calvet, Andrew K. Godwin, Roy A. Jensen, Xiaogang Li

**Affiliations:** 1 Department of Internal Medicine, University of Kansas Medical Center, Kansas City, KS 66160 USA; 2 Department of Biochemistry and Molecular Biology, University of Kansas Medical Center, Kansas City, KS 66160 USA; 3 Department of Cancer Biology, University of Kansas Medical Center, Kansas City, KS 66160 USA; 4 Department of Pathology & Laboratory Medicine, University of Kansas Medical Center, Kansas City, KS 66160 USA

## Abstract

We identified SMYD2, a SMYD (SET and MYND domain) family protein with lysine methyltransferase activity, as a novel breast cancer oncogene. SMYD2 was expressed at significantly higher levels in breast cancer cell lines and in breast tumor tissues. Silencing of SMYD2 by RNAi in triple-negative breast cancer (TNBC) cell lines or inhibition of SMYD2 with its specific inhibitor, AZ505, significantly reduced tumor growth in vivo. SMYD2 executes this activity via methylation and activation of its novel non-histone substrates, including STAT3 and the p65 subunit of NF-κB, leading to increased TNBC cell proliferation and survival. There are cross-talk and synergistic effects among SMYD2, STAT3, and NF-κB in TNBC cells, in that STAT3 can contribute to the modification of NF-κB p65 subunit post-translationally by recruitment of SMYD2, whereas the p65 subunit of NF-κB can also contribute to the modification of STAT3 post-translationally by recruitment of SMYD2, leading to methylation and activation of STAT3 and p65 in these cells. The expression of SMYD2 can be upregulated by IL-6-STAT3 and TNFα-NF-κB signaling, which integrates epigenetic regulation to inflammation in TNBC development. In addition, we have identified a novel SMYD2 transcriptional target gene, PTPN13, which links SMYD2 to other known breast cancer associated signaling pathways, including ERK, mTOR, and Akt signaling via PTPN13 mediated phosphorylation.

## Introduction

Triple-negative breast cancer (TNBC), in which the expression of estrogen receptor (ER), progesterone receptor and human epidermal growth factor receptor 2 are lacking, is a common and aggressive subtype of breast cancer with poor prognostic outcome and reduced short-term survival compared with other types of breast cancer^1^. Due to the loss of three important receptors, TNBC is more difficult to treat and more likely to recur. The challenges of TNBC are in fact more fundamental than insensitivity to current available therapeutics. A major barrier to developing TNBC therapies is our lack of understanding of the molecular drivers of TNBC. As a result, the roles of epigenetic modulation of gene expression and protein function in breast cancer have become a major focus of scientific investigation^2–4^. Identifying the epigenetic signaling networks whose dysregulation drives TNBC would have an enormous impact on our understanding of the disease and how we treat patients.

In eukaryotic cells, genomic DNA is densely packed with histones to form chromatin. Active transcription requires local unwinding of the chromatin structure with post-translational modifications of histones to facilitate accessibility of transcription factors. Histone lysine methylation can occur at particular lysines of histone H3 and H4 to either activate or repress transcription. The accumulated evidence suggests that many histone/lysine methyltransferases function as oncogenes or tumor-suppressors to regulate cancer initiation and progression^5–7^. A SET and MYND domain-containing histone (lysine) methyltransferase, SMYD2, methylates histone H3K4 and H3K36 and non-histone breast cancer associated proteins, including p53, Rb, HSP90 and estrogen receptor α (ERα)^8–13^. SMYD2 methylates p53 to prevent p53 from binding to its target gene promoters, and knockdown of SMYD2 enhances DNA damage-induced, p53-dependent apoptosis^10^. SMYD2 methylates Rb on lysines, which results in either the repression of specific Rb/E2F genes or an increase in Rb phosphorylation, leading to cell cycle progression^11^. Under estrogen-depleted conditions, SMYD2 methylates ERα to prevent its recruitment to its target gene promoters^13^. SMYD2 has been reported to be overexpressed in esophageal squamous cell carcinoma (ESCC) primary tumor samples and in pediatric acute lymphoblastic leukemia correlated with a poor prognosis and patient survival^14,15^. Genetic knockdown of SMYD2 leads to decreased ESCC cell proliferation via cell cycle regulation and apoptosis^14^. Quantitative reverse transcription PCR (qRT-PCR) analysis indicated that SMYD2 mRNA levels in 14 out of 20 breast cancer cell lines were increased at least two-fold compared to those in MCF10A cells, an immortalized but non-tumorigenic breast epithelial cell line^4^. However, the roles and mechanisms by which SMYD2 promotes cancer progression remain unknown.

In this study, we found that SMYD2 promotes triple-negative breast cancer development via the synergistic methylation and activation of its specific non-histone substrates, STAT3 and NF-κB, and via the methylation of histones to transcriptionally regulate the expression of gene(s) related to cancer development. We also found that knockdown of SMYD2 and inhibition of SMYD2 with its specific inhibitor, AZ505, prevented tumor growth in TNBC cells implanted nude mice. Understanding the roles and mechanisms of SMYD2 in TNBC should make SMYD2 an attractive drug target for TNBC treatment, which lacks specific targeted therapy options at present.

## Results

### SMYD2 is highly expressed in triple negative breast cancers

**Fig. 1:**
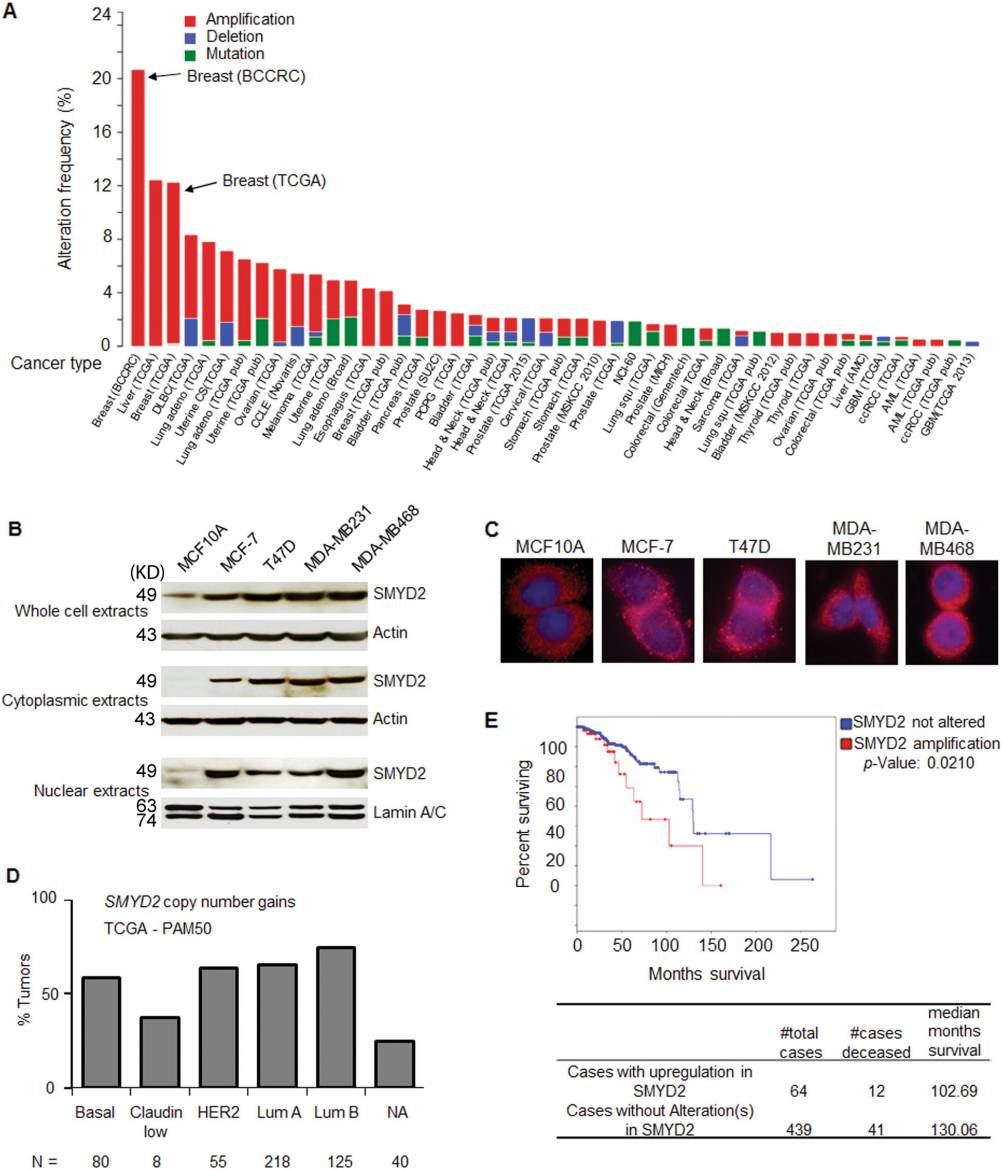
SMYD2 is highly expressed in triple-negative breast cancers. **a** The expression profiles of SMYD2 were analyzed in cross-cancer alteration from 91 studies with cBio Cancer Genomics Portal. SMYD2 was upregulated in all types of cancers, particularly in breast cancers. **b** Western blot analysis of SMYD2 expression from whole cell lysates, cytoplasmic fraction and nuclear fraction in human breast cancer cells as well as in non-tumorigenic MCF10A mammary epithelial cells. **c** Immunofluorescence staining of SMYD2 in human breast cancer cells as well as in MCF10A cells. **d** and **e** The upregulation of SMYD2 was found in all subtypes of breast cancers and was highly related to poor survival

The genetic alterations of histone methyltransferases, including SMYD2, in breast cancer were systematically investigated in breast cancer samples from the cancer genome atlas (TCGA) database via cBio Portal^16,17^. We found that SMYD2 was upregulated in almost all cancer types based on the cross-cancer alteration summary for SMYD2, which included 91 studies, and this was particularly true in breast cancer samples (Fig. 1a). And the protein levels of SMYD2 were increased in two TNBC cell lines, MDA-MB231, MDA-MB468 cells as well as in MCF-7 cells and T47D cells, compared to MCF10A cells in both cytoplasmic fraction and nuclear fraction (Fig. 1b). We also found that SMYD2 was localized to both the cytoplasm and nucleus in TNBC cells. (Fig. 1c).In addition, significantly elevated SMYD2 copy number was found in all subtypes of breast cancer and increased SMYD2 levels were correlated with poor patient survival (Figs. 1d and e). In the basal-like breast cancer subtype, compared with non-basal subtypes, the expression levels of SMYD2 were significantly higher (*p* < 0.001). For SMYD3, which shares a high degree of sequence homology with SMYD2, the expression levels were significantly lower (*p* < 0.001)^4^.

### Knockdown of SMYD2 decreased tumor growth in mice bearing xenografts of TNBC cells

**Fig. 2:**
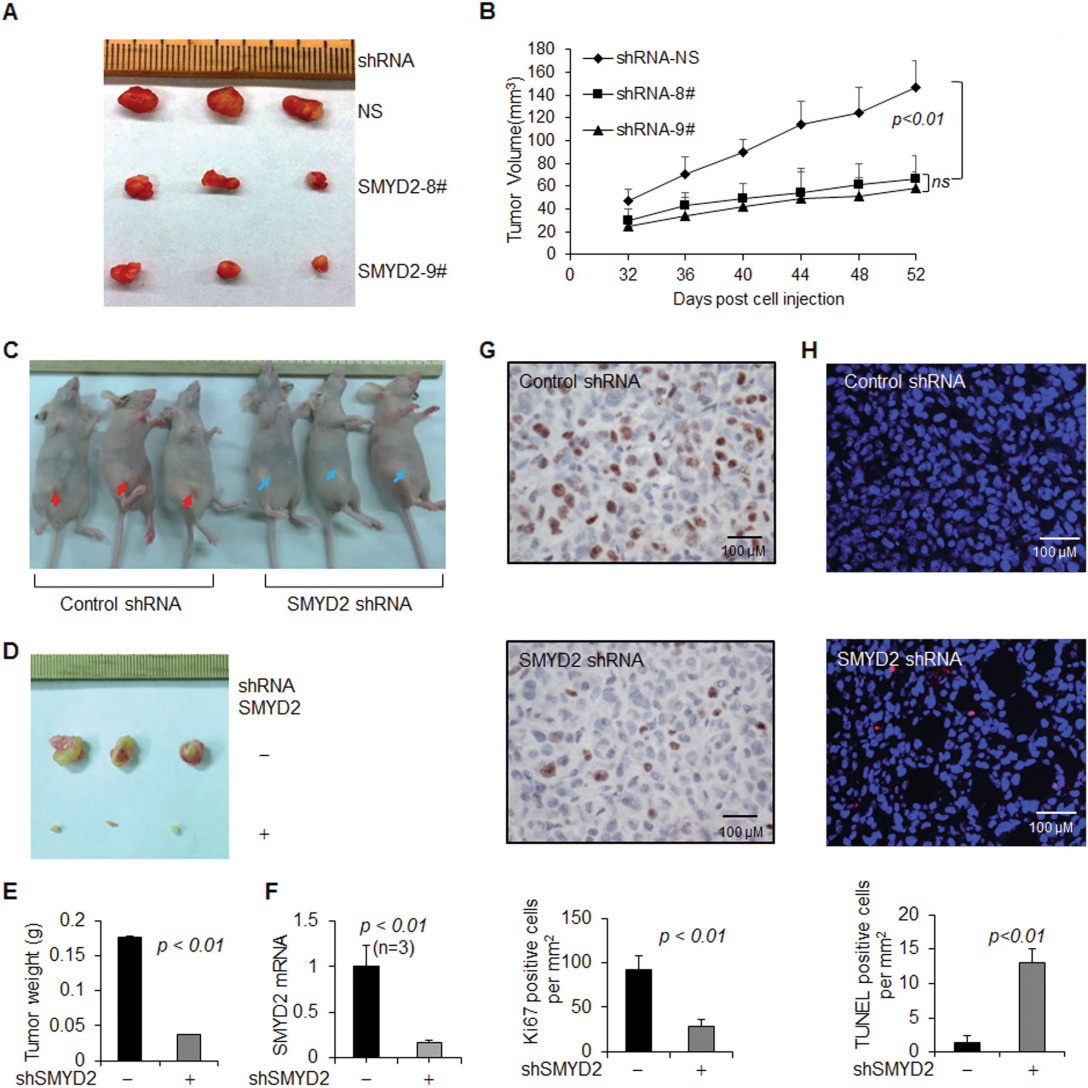
Knockdown of SMYD2 delays tumor growth in triple negative breast cancer cells implanted nude mice. **a** Image depicting the reduction in tumor size observed when SMYD2-shRNA transfected MDA-MB468 cells were injected subcutaneously into mice compared with control. **b** Tumor growth curve in female athymic nude mice bearing MDA-MB468 tumor xenografts post implantation of 5 × 10^6^ with either shRNA vehicle or shRNA-SMYD2-#8 or shRNA-SMYD2-#9—MDA-MB468 human breast cancer cells. Each point represents mean ± SD, *n* = 6. **c–e** Images (**c** and **d**) and graph (**e**) depicting the reduction in tumor size and tumor weight observed when SMYD2-shRNA transfected MDA-MB231 cells that were injected subcutaneously into mice compared with control. Unpaired *t*-test was performed on tumor weight, *p* < 0.01, *n* = 6. **f** qRT-PCR analysis indicated that SMYD2 mRNA levels were almost undetectable in SMYD2 knockdown tissues (*n* = 3). **g** Tumor cell proliferation was decreased in SMYD2-shRNA transfected MDA-MB231 cells xenograft nude mice compared with control shRNA transfected MDA-MB231 cells xenograft nude mice, as examined with Ki67 staining. The percentage of Ki67-positive nuclei was calculated in per mm^2^. *p* < 0.01. Scale bars, 100 μm. **h** Knockdown of SMYD2 induced tumor cell death in MDA-MB231 cells xenografts nude mice compared with controls, as examined by TUNEL assay. *p* < 0.01. Scale bars, 100 μm

To investigate whether SMYD2 is required for in vivo tumor growth, SMYD2 shRNA stable knockdown TNBC cells were implanted in nude mice and tumor growth was measured and compared with that in mice implanted with control vector-infected TNBC parental cells. Robust tumors developed in mice implanted with the control TNBC cells within 30 days. In contrast, tumor growth was inhibited in two SMYD2 knockdown MDA-MB468 cells (Figs. 2a, b) and MDA MB231 cells (Figs. 2c–e) as seen by a decrease in tumor sizes compared to the control mice. We also confirmed the knockdown of SMYD2 in tissues by qRT-PCR (Fig. 2f). We also found that depletion of SMYD2 decreased tumor cell proliferation assayed by Ki67 staining (Fig. 2g) and induced apoptosis by TUNEL assay (Fig. 2h). In addition, we found that overexpressing GFP-tagged SMYD2 increased cell proliferation of HEK293T cells and MCF10A cells (Supplementary Fig. S1A and 1B). These results suggested that SMYD2 functions as a regulator of tumor progression in TNBC.

### Inhibition of SMYD2 with AZ505 suppressed tumor growth in mice bearing xenografts of TNBC cells

**Fig. 3:**
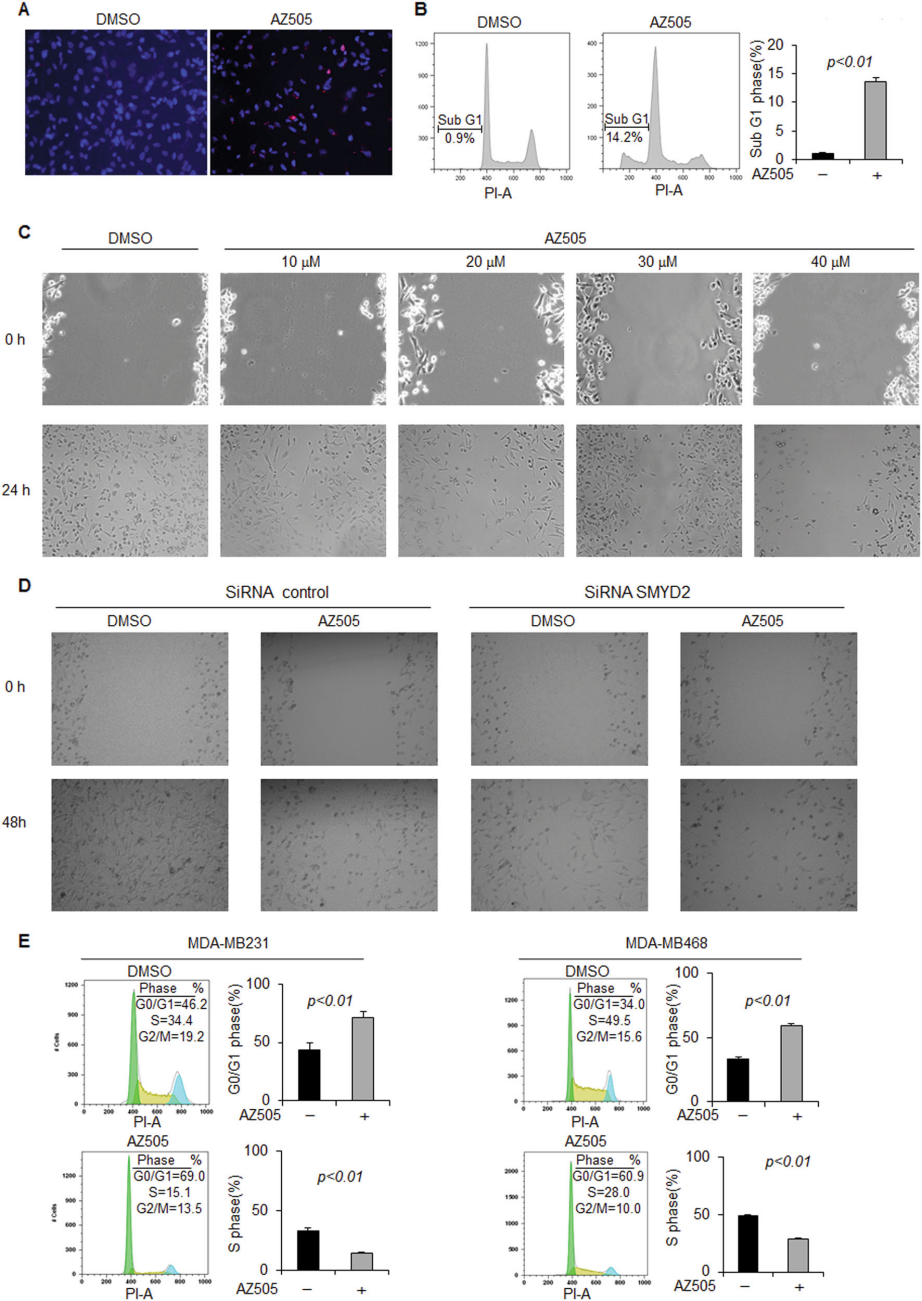

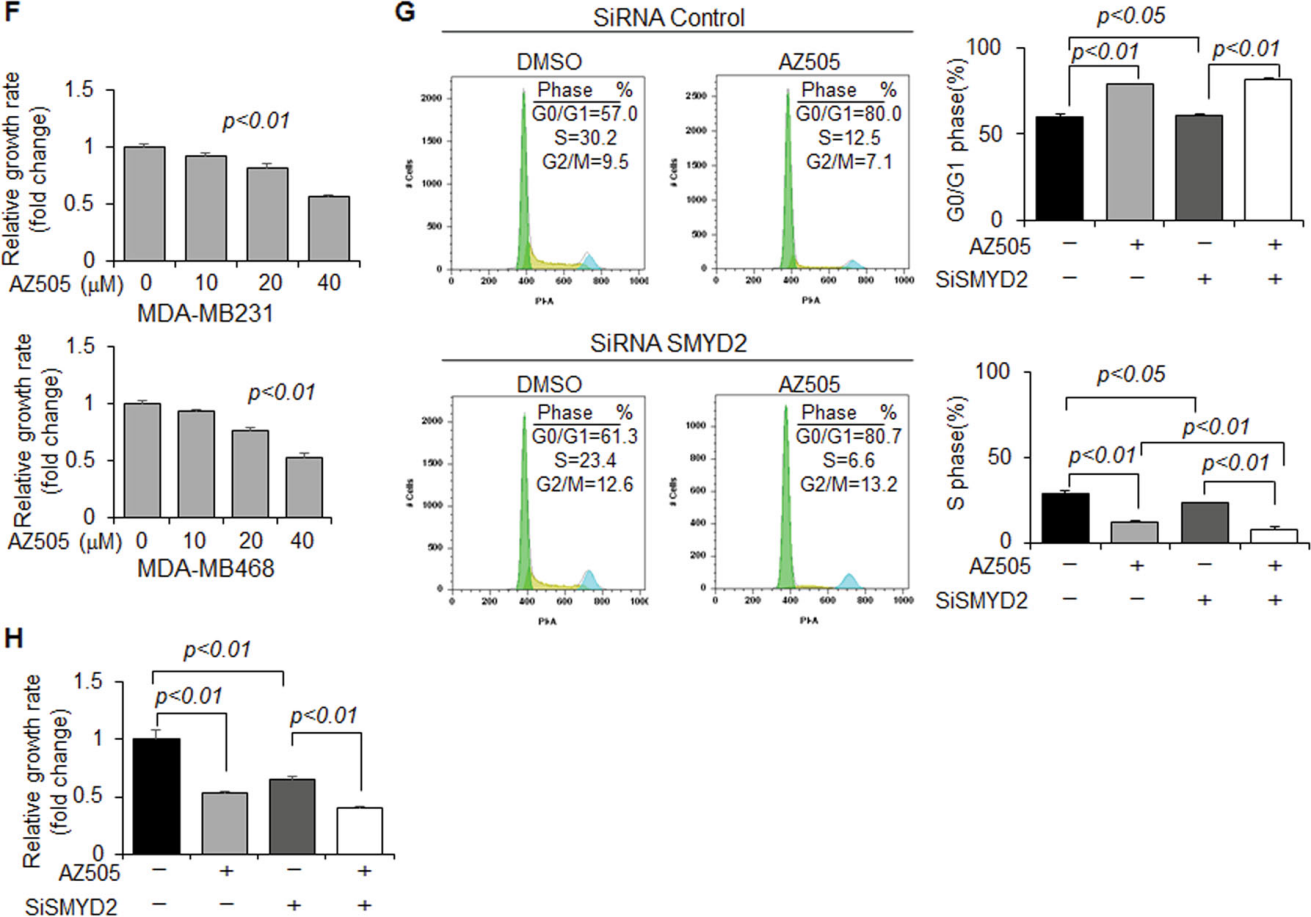
Inhibition of SMYD2 with its specific inhibitor, AZ505, induced TNBC cell apoptosis but suppressed TNBC cell migration and proliferation in vitro. **a** AZ505 treatment (20 μM, 24 h) induced MDA-MB231 cell death, as examined by TUNEL assay. One representative experiment from two independent experiments with similar results was shown. **b** AZ505 treatment (20 μM, 24 h) increased Sub-G1 phase in MDA-MB468 cells as examined by FACS analysis. *n* = 3. **c** Wound closure assays indicated that AZ505 treatment (24 h) suppressed MDA-MB231 cell migration in a dose dependent manner. **d** Would closure assays indicated that knockdown of SMYD2 suppressed MDA-MB231 cell migration, and AZ505 treatment further suppressed these cell migration in SMYD2-knockdown MDA-MB231 cells, 20 μM AZ505 was added in the medium 24 h after siRNA or control was transfected, then cells were incubated for another 24 h. **e** FACS analysis indicated that inhibition of SMYD2 with AZ505 induced cell-cycle arrest at the G0/G1 phase in MDA-MB231 cells (left panel) and MDA-MB468 cells (right panel). **f** Inhibition of SMYD2 with AZ505 inhibited the proliferation of both MDA-MB231 cells and MDA-MB468 cells in a dose dependent manner as examined by MTT assay. The AZ505 was added according to the concentrations shown in the figure for 24 h. **g** FACS analysis indicated that knockdown of SMYD2 with siRNA induced cell-cycle arrest at the G0/G1 phase in MDA-MB231 cells, and treatment with AZ505 further induced the cell cycle arrest in SMYD2-knockdown MDA-MB231 cells. One of three independent experiments was shown. **h** Knockdown of SMYD2 inhibited MDA-MB231 cell proliferation as indicated by MTT assay. Treatment with AZ505 only slightly further decreased the proliferation of SMYD2 knockdown MDA-MB231 cells

AZ505 was discovered through a high throughput chemical screen as a selective inhibitor of SMYD2 when it was found to bind the substrates binding groove of SMYD2^18^. We found that AZ505 treatment induced apoptosis compared to DMSO treated cells as measured by TUNEL and FACS analysis (Figs. 3a, b). We also found that AZ505 treatment inhibited MDA-MB231 cell migration determined in a wound closure assay (Fig. 3c), and treatment with AZ505 just slightly further inhibited the cell migration in SMYD2-knockdown MDA-MB231cells (Fig. 3d). In addition, we found AZ505 treatment decreased S-phase entry in both MDA-MB231 and MDA-MB468 cells as examined by FACS analysis (Fig. 3e). The effect of AZ505 on TNBC cell proliferation is in a dose dependent manner as examined by MTT assay (Fig. 3f). Similar inhibition effects of AZ505 on cell proliferation and cell migration were also found in ER positive MCF-7 cells and T47D cells (Supplementary Fig. S2). Knockdown of SMYD2 also inhibited cell cycle in MDA-MB231 cells, while treatment with AZ505 further inhibited cell cycle in SMYD2-knockdown cells (Fig. 3g). Treatment with AZ505 further inhibited cell proliferation in SMYD2 knockdown MDA-MB231 cells compared to that in the DMSO treated SMYD2 knockdown cells (Fig. 3h). The results shown in Figs. 3d, g, and h might be caused by the inhibition of AZ505 on the remaining SMYD2 in SMYD2 knockdown cells since we found that treatment with AZ505 didn’t further affect cell proliferation of Smyd2 knockout cells (Supplementary Fig. S1,C). These results support the specificity of AZ505 on the inhibition of SMYD2.

**Fig. 4:**
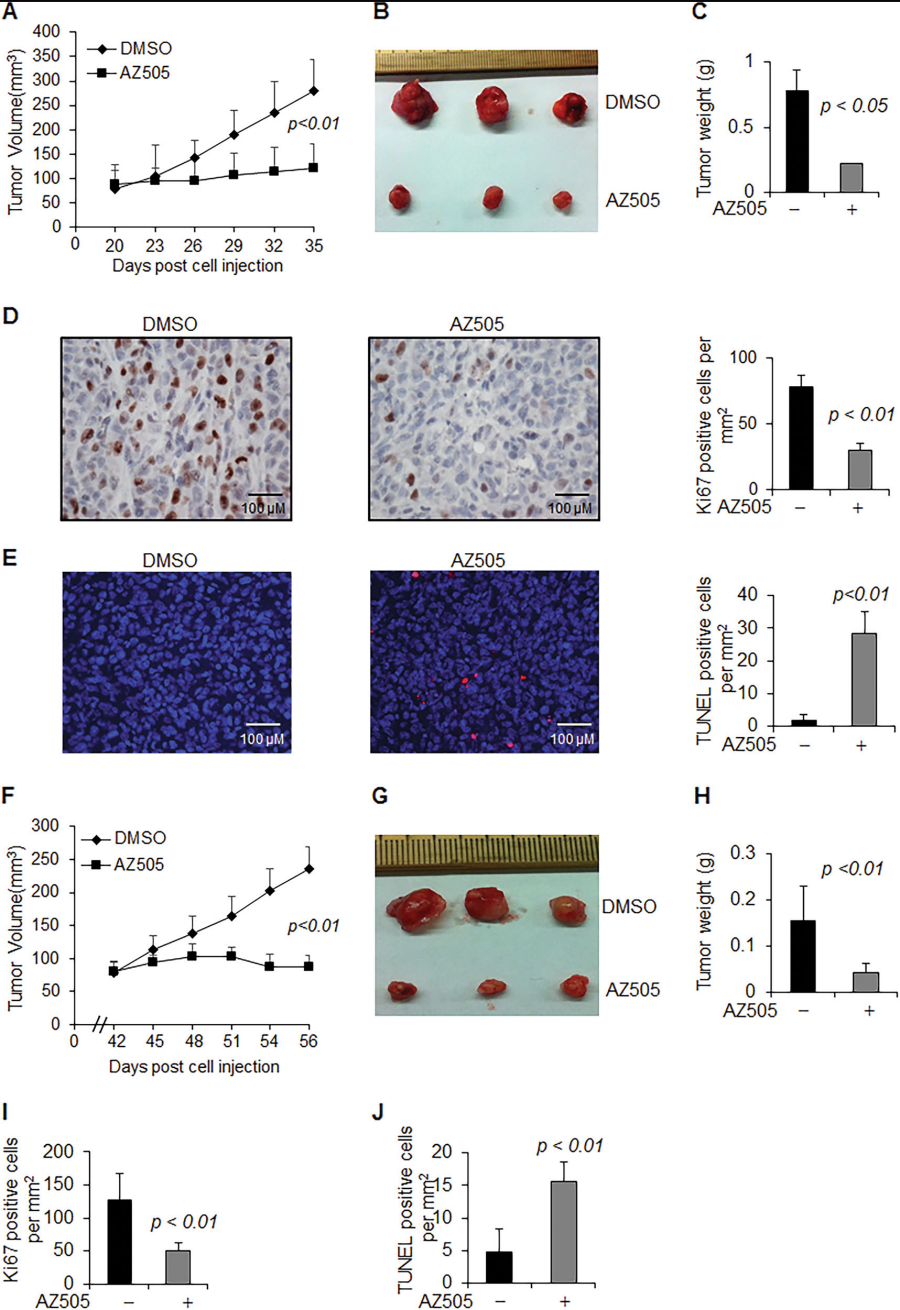
Inhibition of SMYD2 with its specific inhibitor, AZ505, delays tumor growth in vivo. **a** Tumor growth inhibition curve in female athymic nude mice bearing MDA-MB231 tumor xenografts treated intraperitoneally with either vehicle or AZ505 at doses described in Materials and Methods for fourteen consecutive days beginning on day 21 post implantation of 5 × 10^6^ MDA-MB231 human breast cancer cells. Each point represents mean ± SD, *n* = 10. **b** and **c** Image (**b**) and graph (**c**) depicting the reduction in tumor size and tumor weight observed when MDA-MB231 implanted mice were treated with AZ505 compared to vehicle DMSO treated controls. Unpaired *T*-test was performed on tumor weight, *p*-value < 0.01, *n* = 10. **d** Tumor cell proliferation was decreased in xenografts from MDA-MB231 implanted mice treated with AZ505 compared to those treated with vehicle DMSO, as examined with Ki67 staining. The percentage of Ki67-positive nuclei was calculated in per mm^2^. *p*-value < 0.01. Scale bars, 100 μm. **e** AZ505 treatment induced tumor cell death in xenografts from MDA-MB231 implanted mice compared to those mice treated with vehicle DMSO, as examined by TUNEL assay. *p* < 0.01. Scale bars, 100 μm. **f** Tumor growth inhibition curve in female athymic nude mice bearing MDA-MB468 tumor xenografts treated intraperitoneally with either vehicle or AZ505 at doses described in Materials and Methods for fourteen consecutive days beginning on day 42 post implantation of 5 × 10^6^ MDA-MB468 human breast cancer cells. Each point represents mean ± SD, *n* = 5. **g** and **h** Images (**g**) and graph (**h**) depicting the reduction in tumor size and tumor weight observed when MDA-MB468 implanted mice are treated with AZ505 compared to vehicle DMSO treated controls. Unpaired *T*-test was performed on tumor weight, *p* < 0.01, *n* = 5. **i** Tumor cell proliferation was decreased in xenografts from MDA-MB468 implanted mice treated with AZ505 compared to those treated with vehicle DMSO, as examined with Ki67 staining. The percentage of Ki67-positive nuclei was calculated in per mm^2^. *p* < 0.01. **j** AZ505 treatment induced tumor cell death in xenografts from MDA-MB468 implanted mice treated with AZ505 compared to those mice treated with vehicle DMSO, as detected by TUNEL assay. *p* < 0.01

We further found that the tumor growth in MDA-MB231 implanted female nude mice was significantly retarded by daily intraperitoneal injections of AZ505 at a dose of 40 mg/kg body weight (Figs. 4a–c). AZ505 treatment also decreased tumor cell proliferation and induced tumor cell apoptosis as seen by Ki67 staining (Fig. 4d) and TUNEL analysis (Fig. 4e). The effect of AZ505 on inhibition of TNBC growth was also confirmed in nude mice with MDA-MB468 xenografts (Figs. 4f–j). Our results suggest that SMYD2 may serve as a potent therapeutic target in TNBC treatment justifying further investigation into the mechanisms of SMYD2 in TNBC development.

### SMYD2 interacts with STAT3 and the p65 subunit of NF-κB in TNBC cells

**Fig. 5:**
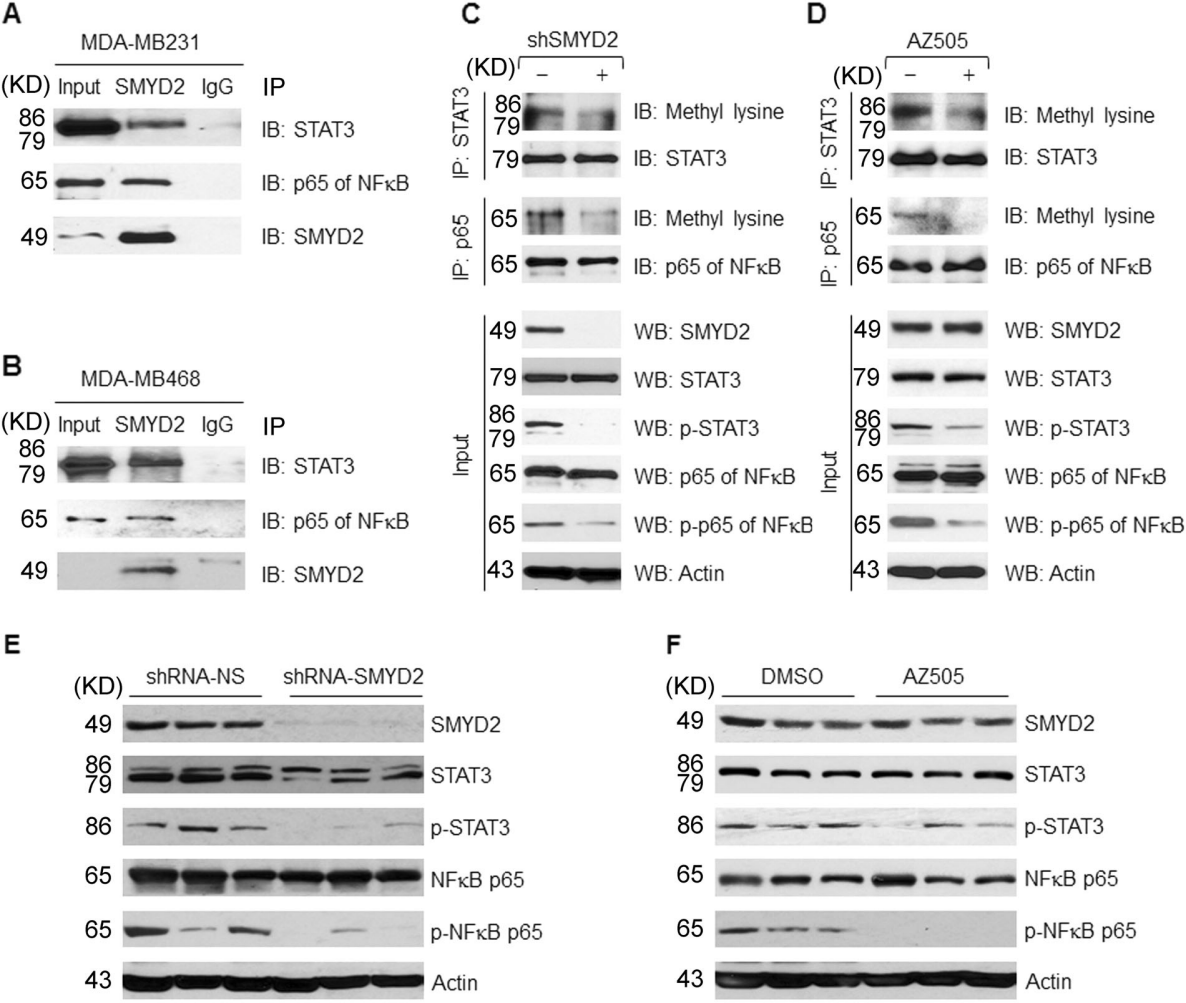
SMYD2 interacts and methylates STAT3 and the p65 subunit of NF-κB, resulting in the phosphorylation and activation of STAT3 and p65 in TNBC cells. **a** and **b** Interactions between SMYD2 and STAT3 as well as SMYD2 and p65 in MDA-MB231 cells and MDA-MB468 cells were examined by immunoprecipitation with anti-SMYD2 antibody and then blotted with STAT3 antibody and p65 antibody, respectively. IgG was used as a negative control. **c** and **d** Western blot analysis of the methylation of STAT3 and p65 in MDA-MB231 cells. Knockdown of SMYD2 with shRNA (**c**) or inhibition of SMYD2 with AZ505 (40 μM, 2 h) (**d**) decreased the methylation and phosphorylation of STAT3 and p65, but did not affect their expression in MDA-MB231 cells. Representative result from at least three independent experiments was shown. **e** and **f** Western blot analysis of the phosphorylation of STAT3 and p65 in MDA-MB231 bearing xenograft. Knockdown of SMYD2 with shRNA (**e**) or inhibition of SMYD2 with AZ505 (**f**) decreased the phosphorylation of STAT3 and p65, but did not affect their expression

Triple-negative breast cancers are associated with activation through an unknown mechanism of the JAK2/STAT3 signaling pathway^19^. Numerous studies have shown that NF-κB activation in tumors mimics a classical inflammatory response^20–22^ and appears to result from upregulation of cytokine gene expression in breast cancer cells. We found that SMYD2 interacts with STAT3 and the p65 subunit of NF-κB in both MDA-MB231 and MDA-MB468 cells (Figs. 5a, b).

### SMYD2 regulates the methylation and phosphorylation of STAT3 and the p65 subunit of NF-κB in TNBC cells

To determine if SMYD2 is involved in methylation of STAT3 and p65, leading to their activation in TNBC cells, we examined whether shRNA knockdown of SMYD2 decreased the levels of methylated STAT3 and p65 in MDA-MB231 and MDA-MB468 cells. Methylation was assessed with an anti-pan methylated lysine antibody. An anti-STAT3 antibody and an anti-p65 antibody were used to pull down STAT3 and p65, which were then blotted with an anti-pan methylated lysine antibody, and the same blot was then stripped and re-probed with the anti-STAT3 and anti-p65 antibodies to confirm their presence. We found that silencing of SMYD2 with shRNA decreased the methylation of STAT3 and p65 in MDA-MB231 (Fig. 5c) and MDA-MB468 cells (Supplementary Fig. S3A). Knockdown of SMYD2 with shRNA also decreased the phosphorylation of STAT3^Y705^ and p65^S536^ while total STAT3 and p65 expression levels were not changed. However, overexpression of SMYD2 increased the methylation and phosphorylation of STAT3^Y705^ and p65^S536^ in HEK293T cells (Supplementary Fig. S3B). We also found that treatment with AZ505 decreased the methylation and phosphorylation of STAT3 and p65 in MDA-MB231 (Fig. 5d) and MDA-MB468 cells (Supplementary Fig. S3C). The phosphorylation of STAT3 and p65 were also decreased in tumors from SMYD2 knockdown (Fig. 5e) and AZ505 treated MDA-MB231 bearing mice (Fig. 5f). Our in vitro methylation assay confirmed that SMYD2 could methylate STAT3 and p65 at K685 and K310, respectively (Supplementary Fig. S4A and S4B). These results suggested that SMYD2 methylation of STAT3 and p65 contributes to the hyper-phosphorylation of STAT3 and p65 in MDA-MB231 and MDA-MB468 cells, which promotes tumor growth^23,24^.

### There is a synergistic effect between SMYD2, STAT3, and NF-κB p65 in TNBC cells

**Fig. 6:**
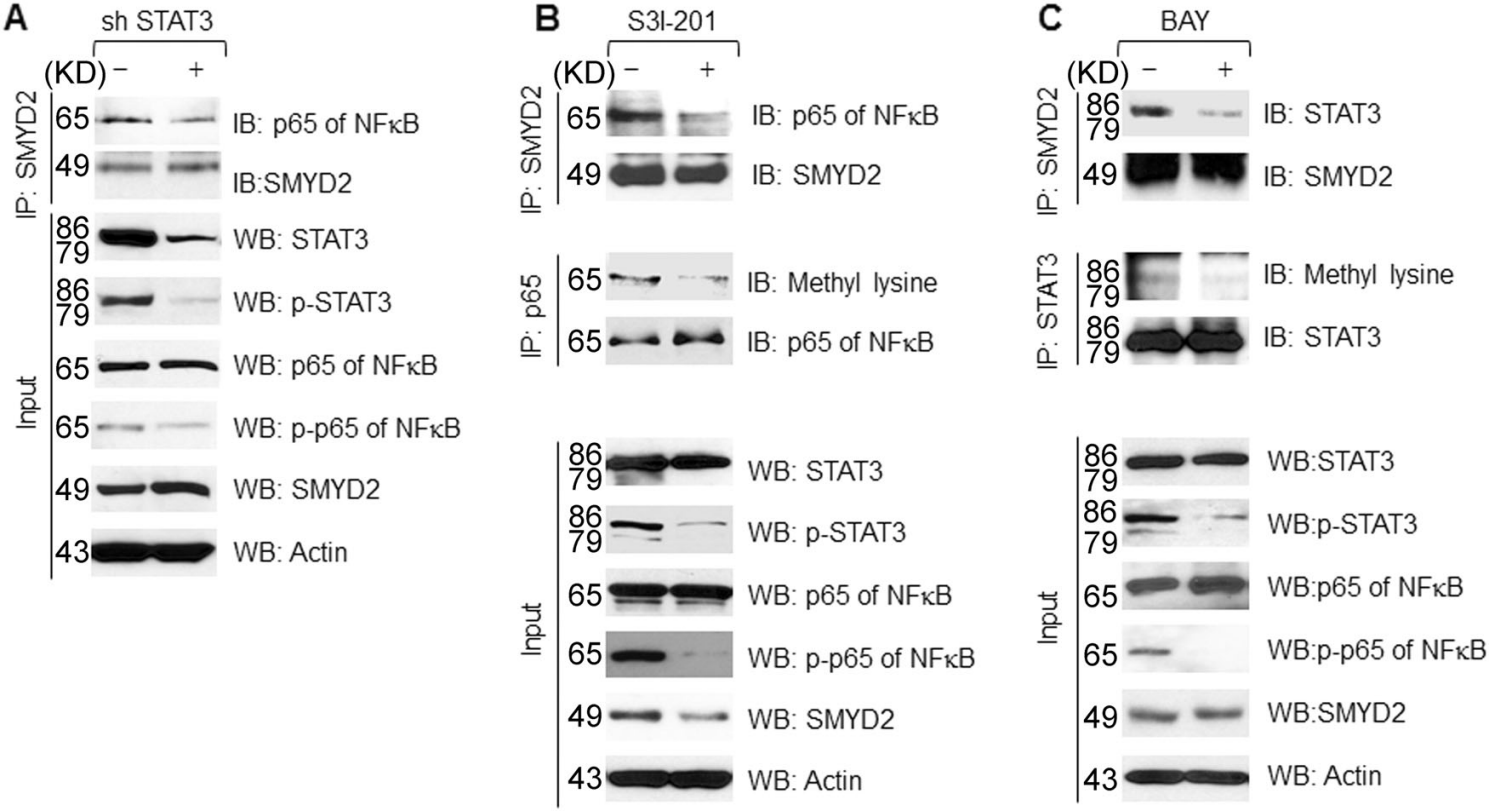
Synergistic effects exist among SMYD2, STAT3 and NF-κB in MDA-MB231 cells. **a** Knockdown of STAT3 disrupted the interaction between SMYD2 and p65 and decreased the phosphorylation of p65 in MDA-MB231 cells. **b** Inhibition of STAT3 with its inhibitor S3I-201(100 μM, 3 h) disrupted the interaction between SMYD2 and p65, and decreased the methylation and phosphorylation of p65 as well as the expression of SMYD2 in MDA-MB231 cells. Representative result from at least two independent experiments was shown. **c** Inhibition of NFκB with its inhibitor BAY-11-7085(40 μM, 3 h) disrupted the interaction between SMYD2 and STAT3, and decreased the methylation and phosphorylation of STAT3 in MDA-MB231 cells. Representative result from at least two independent experiments was shown

STAT3 can modify p65 post-translationally by recruitment of the acetyltransferase p300 to mediate the acetylation of NF-κB to prolong its nuclear retention^25^. To address the role of STAT3 in SMYD2 mediated NF-κB activation, we treated TNBC cells with STAT3 shRNA and S3I-201, a commonly used STAT3 inhibitor that binds to the STAT3-SH2 domain to prevent STAT3 phosphorylation, dimerization, and DNA binding^26^. We found that knockdown of STAT3 and inhibition of STAT3 with S3I-201 not only disrupted the interaction between SMYD2 and p65 (Figs. 6a, b, top panel) but also decreased the methylation of p65 as well as the phosphorylation of STAT3^Y705^ and p65^S536^ in MDA-MB231 cells (Figs. 6a, b). Treatment with the NF-κB inhibitor, BAY-11-7085, also disrupted the interaction between SMYD2 and STAT3 and decreased the methylation and phosphorylation of STAT3 as well as the phosphorylation of p65 (Fig. 6c). Based on this, we proposed that STAT3 contributes to SMYD2 mediated NF-κB activation and that p65 also contributes to SMYD2 mediated STAT3 activation.

### There are feedback networks among SMYD2, STAT3, NF-κB, and cytokines in TNBC cells

**Fig. 7:**
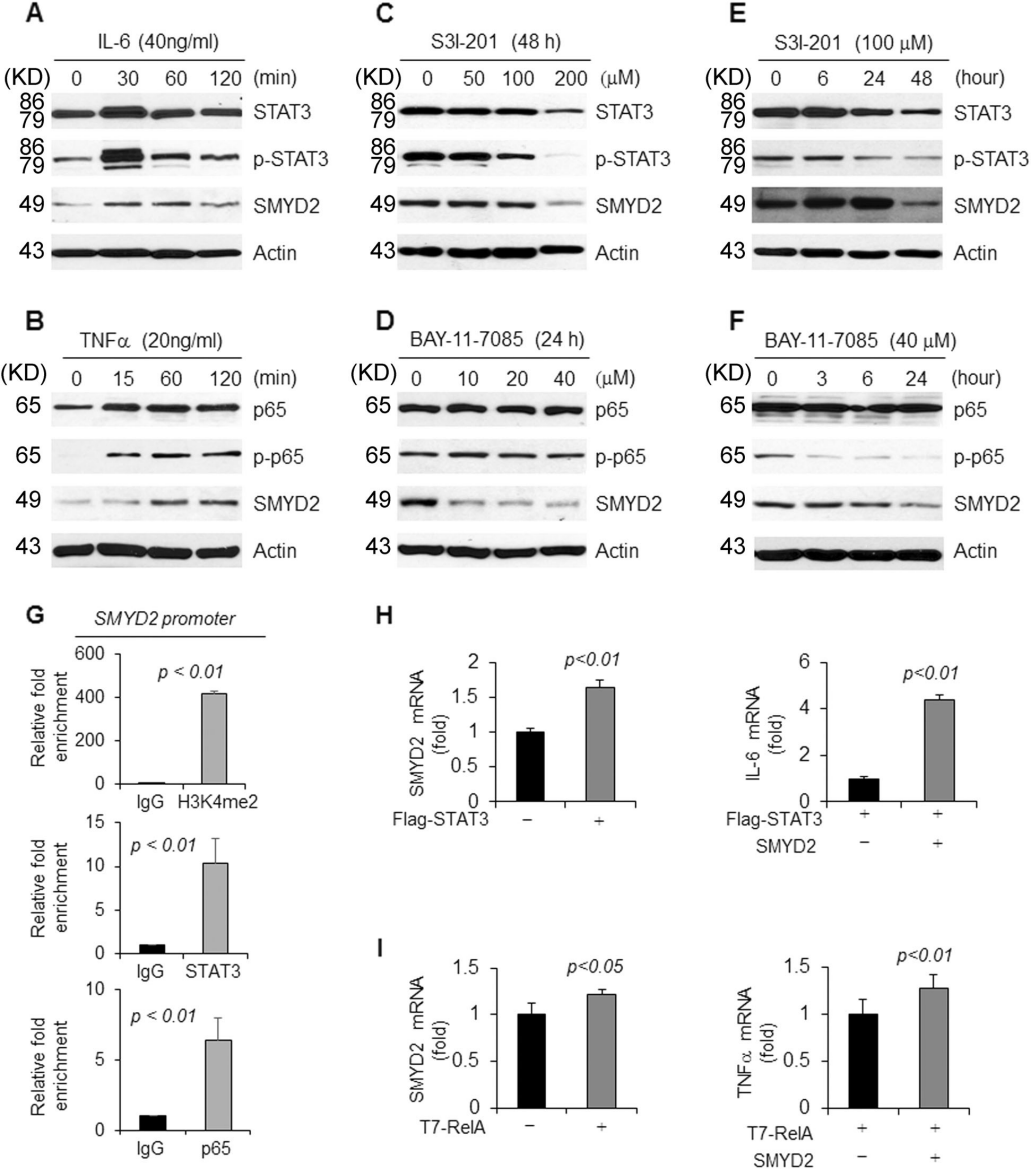
Feedback loops exist in TNBCs to regulate the expression of SMYD2. **a** and **b** Stimulation with cytokines, IL-6 (**a**) and TNF-α (**b**), induced SMYD2 expression in a time dependent manner in MCF10A cells. **c** and **d** Western blot analysis of SMYD2 expression in MDA-MB468 cells treated with STAT3 inhibitor S3I-201 (**c**) or NF-κB inhibitor BAY-11-7085 (**d**). SMYD2 expression was decreased in MDA-MB468 cells treated with these inhibitors in a dose dependent manner. **e** and **f** Western blot analysis of SMYD2 expression in MDA-MB468 cells treated with STAT3 inhibitor S3I-201 (**e**) or NFκB inhibitor BAY-11-7085 (**f**). SMYD2 expression was decreased in MDA-MB468 cells treated with STAT3 and NFκB inhibitors in a time dependent manner. **g** STAT3 and p65 bound to the promoter of SMYD2 as examined by ChIP-qPCR. ChIP assay was performed with anti-STAT3, anti-p65 antibody or normal rabbit IgG in MDA-MB231 cells. Anti-H3K4me2 antibody was used as a positive control. **h** Overexpression of Flag-tagged STAT3 upregulated the expression of SMYD2 mRNA (left panel), and coexpression of STAT3 and SMYD2 increased the expression of IL-6 mRNA (right panel). **i** Overexpression of T7-tagged RelA (p65) upregulated the expression of SMYD2 mRNA (left panel), and coexpression of p65 and SMYD2 increased the expression of TNF-α mRNA (right panel)

Both STAT3 and NF-κB p65 are important transcription factors, which regulate gene expression involved in cell proliferation and apoptosis through binding directly to promoters. These two proteins also affect cellular signaling pathways indirectly through their downstream cytokines, in which IL-6 and TNFα have played particularly important roles, because they can form a feedback loop with STAT3 and NF-κB, respectively^27,28^. We found that IL-6 treatment not only increased the phosphorylation of STAT3 but also increased the expression of SMYD2 in MCF-10A cells (Fig. 7a), whereas treatment with TNFα also increased the phosphorylation of p65 and the expression of SMYD2 in these cells (Fig. 7b). We also found that treatment with the STAT3 inhibitor, S3I-201 and the NF-κB inhibitor, BAY-11-7085, decreased the expression of SMYD2 in these cells in a dose dependent (Figs. 7c, d) and time dependent (Figs. 7e, f) manner. We further found that STAT3 and p65 bound to the promoter of SMYD2 in MDA-MB231 cells (Fig. 7g). In addition, overexpression of STAT3 and p65 increased the expression of SMYD2 mRNA, and coexpression of STAT3 or p65 with SMYD2 increased the expression of IL-6 and TNFα, respectively (Figs. 7h, i). These results suggest the existing of two positive feedback loops in TNBC cells: SMYD2-IL-6-STAT3-SMYD2 and SMYD2-TNFα-NF-κB-SMYD2.

### SMYD2 regulates other signaling pathways in TNBC cells via the methylation of histones

Our previous ChIP-seq data indicated that PTPN13 was regulated by SMYD2 via the methylation of histones^29^, which regulates a variety of cellular processes and oncogenic transformation by removing phosphate groups from phosphorylated tyrosine residues on a number of proteins, including ERK and STAT^30,31^. SMYD2 is able to downregulate the expression of PTPN13 in TNBC cells (Supplementary Fig. S5A-S5C), suggesting that SMYD2 mediated downregulation of PTPN13 might be one of the mechanisms responsible for the phosphorylation and activation of these TNBC associated signaling pathways in TNBC cells (Supplementary Fig. S5D and S5E).

## Discussion

The term TNBC came into vogue shortly after the introduction of routine HER-2 testing for breast cancer to describe the clinical subset of breast cancer patients whose tumors tested negative for estrogen receptor, progesterone receptor and HER-2 expression^32^. These patients were notable for their relatively poor prognosis and for their lack of a recognized target for molecular-oriented therapy. In the last few years, based on the aberrant signal transduction pathways that may be involved in regulating growth, survival and the development of chemo-resistance in TNBC, a number of alternative strategies have been evaluated in the advanced clinical stages in TNBC patients^33–35^. However, the clinical results have so far been somewhat disappointing. A clear understanding of the molecular pathology of TNBC, including the pathogenesis of the disease and the mechanisms of resistance to currently available therapies, is a matter of urgency and these efforts should provide novel therapeutic targets for TNBC treatment.

**Fig. 8:**
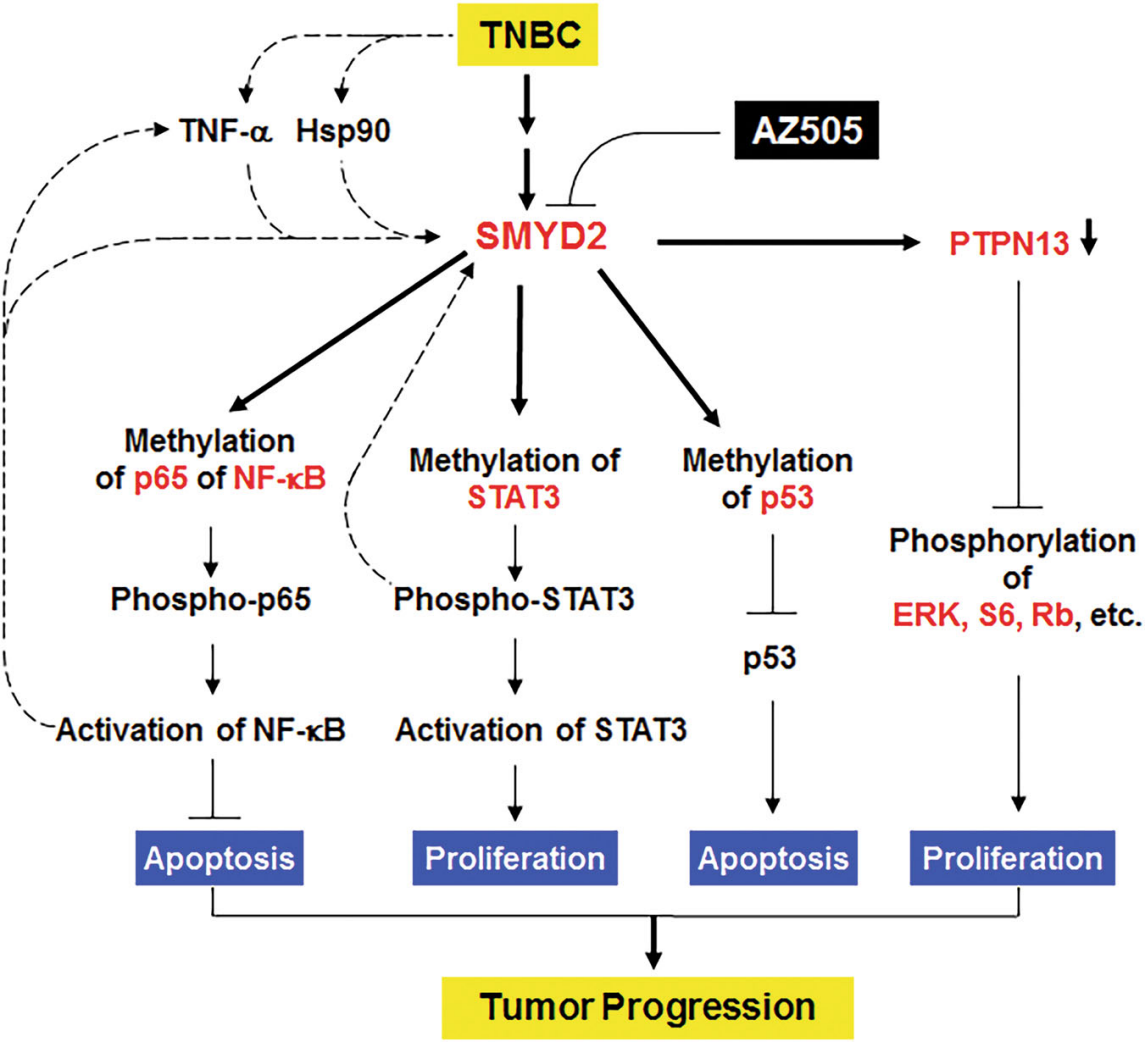
Working model of SMYD2 in regulation of TNBC progression. A schematic diagram depicting SMYD2 mediated pathways and processes in TNBC. SMYD2 was upregulated in TNBC, which may be stabilized via its interaction with HSP90 and induced by TNF-α secreted by immune cells in the tumor microenviroment or through other unknown mechanisms. Upregulated SMYD2 in TNBC cells, (1) methylates STAT3, leading to its activation and tumor cell proliferation; (2) methylates the p65 subunit of NF-κB, leading to its activation which represses tumor cell apoptosis; (3) methylates p53, leading to the repression of p53 and cystic renal epithelial cell apoptosis; and (4) regulates the transcription of PTPN13, a protein of the protein tyrosine phosphatase (PTP) family, which may regulate the phosphorylation and activation of ERK, mTOR, Akt, and RB signaling. Targeting SMYD2 with its specific inhibitor, AZ505, retarded tumor progression in TNBC cell implanted nude mice. In addition, two positive feedback loops can be observed: SMYD2-IL-6-STAT3-SMYD2, and SMYD2-TNF-α-NF-κB-SMYD2, which may further increase the levels of SMYD2 in TNBC cells

In this study, we have demonstrated that SMYD2 is a novel epigenetic regulator associated with TNBC and we have uncovered the underlying mechanisms associated with SMYD2 regulation of TNBC progression (Fig. 8). SMYD2 was found to be significantly expressed at higher levels in TNBC cells both at the mRNA and protein level. We should emphasize that SMYD2 was also upregulated in other types of breast cancer (Fig. 1a). Thus, targeting SMYD2 may also be a potential therapeutic strategy in other types of breast cancers. However, whether the mechanisms identified for SMYD2 in TNBC work in other breast cancer need to be further investigated in the future. In TNBC, we found that upregulation of SMYD2 and the resulting methylation of STAT3 and the p65 subunit of NF-κB lead to their phosphorylation and activation, impacting cell cycle and apoptosis, respectively. We also found a synergistic cross-talk mechanism involving SMYD2, STAT3, and NF-κB in TNBC cells. STAT3 was shown to contribute to the NF-κB p65 subunit post-translational modification by recruitment of SMYD2, leading to the methylation and activation of p65, while the p65 subunit of NF-κB could also contribute to the STAT3 post-translational modification by recruitment of SMYD2, leading to the methylation and activation of STAT3 in TNBC cells. We identified two positive feedback loops, SMYD2-IL-6-STAT3-SMYD2 and SMYD2-TNFα-NF-κB-SMYD2, which integrated epigenetic regulation with inflammation in TNBC development and suggested that SMYD2 could be further induced by immune cell cytokines, such as IL-6 and TNFα, during TNBC progression. In addition, we confirmed that PTPN13 is a SMYD2 transcriptional target gene in TNBC cells, which may link SMYD2 to other breast cancer associated signaling pathways, including ERK, mTOR, and Akt signaling. The crosstalk and synergistic effects between SMYD2 and its non-histone substrates, STAT3 and NF-κB, could be a general mechanism in human diseases that occurs when SMYD2 is upregulated, suggesting that targeting SMYD2 with its specific inhibitor, AZ505, could be a potential strategy for the treatment of TNBC and other diseases in which there is elevation of SMYD2.

Protein-lysine methyltransferases represent one of several families of enzymes with critical roles in epigenetic regulation, as they catalyze the transfer of methyl groups from *S*-adenosyl-L-methionine to acceptor lysine residues on histone and/or non-histone protein substrates, impacting a variety of biological processes and disease states^36,37^. The SMYD family comprises a subset of five SET domain-containing proteins with unique domain architecture, whereas these proteins have different distributions and functions. SMYD1 was reported as regulator of heart and skeletal muscle development^38^. SMYD3 plays a role in transcriptional regulation and tumorigenesis^39^. SMYD4 was identified as a potential tumor suppressor gene involved in breast cancer development^40^. SMYD5 was identified to be critical in cancer metastasis in breast cancer cells during lung colonization^41^. The expression of SMYD3, which shares a high degree of sequence homology with SMYD2, was significantly lower compared to the significantly higher expression of SMYD2 (*p* < 0.001) in basal-like breast tumors^4^. SMYD2 was originally identified as a histone methyltransferase that specifically methylates H3K4 and H3K36 in cooperation with the histone deacetylase complex^8^. Recent studies identified that SMYD2 also methylated key cancer proteins, including RB, p53 and HSP90, ERα, and PTEN, to alter their functions during cancer development^10,11,13,42,43^. In this study, we found that STAT3 and the p65 subunit of NF-κB are novel SMYD2 non-histone substrates, and we provided evidence that upregulation of SMYD2 caused methylation of STAT3 and p65, leading to their phosphorylation and activation, which impacted TNBC cell proliferation and apoptosis.

Cytokine-induced JAK-STAT3 signaling has emerged as a positive regulator of cancer^44^. Our results demonstrated that SMYD2 acts as a novel mediator of STAT3 activation via lysine methylation which results in its phosphorylation. Phosphorylation leads to dimerization and nuclear accumulation of STAT3, which then recognizes target DNAs and activates gene expression^45^. In addition, STAT3 activity is also regulated by the association with multiple cofactors.

It has been shown that the p65 subunit of NF-κB interacts physically with STAT3, facilitating NF-κB recruitment to STAT3 promoters and vice versa, which suggests that NF-κB and STAT3 may cooperatively regulate a number of target genes^46^. We found that the p65 subunit of NF-κB is a novel non-histone substrate of SMYD2 in TNBC cells (Fig. 5). Methylation of p65 via SMYD2 also resulted in its phosphorylation and activation, which might regulate TNBC cell proliferation and survival (Fig. 8). We further found that STAT3 can modify p65 post-translationally by recruitment of SMYD2, mediating the methylation of the NF-κB p65 subunit (Fig. 6). This crosstalk among SMYD2, STAT3, and NF-κB in TNBC may be applied to other human diseases which are characterized by upregulation of SMYD2.

NF-κB, as a transcription factor, can be activated to induce expression of chemokines and cytokines within pre-malignant cells, leading to the recruitment and activation of immune cells. Activated immune cells in turn produce more pro-inflammatory cytokines/chemokines and growth factors, such as interleukin-1 (IL-1), IL-6, and TNFα, in an autocrine and/or paracrine manner to further stimulate NF-κB activation within the pre-malignant cells, forming a positive feedback loop^47^. We found that IL-6 and TNFα treatment not only induced the phosphorylation and activation of STAT3 and p65, respectively, but also increased the expression of SMYD2 (Figs. 7a, b). In addition, treatment with inhibitors of STAT3 and NF-κB decreased the expression of SMYD2 in TNBC cells (Figs. 7c–f), and we further found that STAT3 and p65 bound to the promoter of SMYD2 (Fig. 7g) suggesting that two positive feedback loops exist: SMYD2-IL-6-STAT3-SMYD2 and SMYD2-TNFα-NF-κB-SMYD2 in TNBC cells. NF-κB mediated immune cell activation may produce additional pro-inflammatory cytokines/chemokines and growth factors, including IL-6, and TNFα, in an autocrine and/or paracrine manner during TNBC progression, which may further induce the expression of SMYD2. Thus, our results not only integrate inflammation with epigenetic regulation in TNBC, but also suggest crosstalk between STAT3 and NF-κB in SMYD2 mediated tumor progression. Together, these results suggest that the growth and survival of TNBC cells could be promoted through a synergistic mechanism involving enhanced cytokine signaling and upregulation of SMYD2.

In contrast to the positive feedback between the p65 subunit of NF-κB and STAT3, a mutual inhibition has been reported between p65 and p53, in that the p65 subunit inhibits p53 dependent transactivation and p53 can suppress NF-κB transcriptional activity^48^. It has also been reported that SMYD2 represses p53 activity through methylation^10^. Our results showing that SMYD2 methylated p65 and activated NF-κB suggested another mechanism for the mutual inhibition of p65 and p53, which might be mediated by SMYD2.

Novel therapeutic strategies have now reached advanced stages of clinical evaluation in TNBC patients. However, the only targeted agent currently available for patients with TNBC remains the anti-VEGF monoclonal antibody bevacizumab, which has not been specifically designed for TNBC. Our study provides evidence that deregulation of SMYD2 appears to play a crucial role in TNBC. Our study has shown for the first time the effect of the specific SMYD2 inhibitor, AZ505, in an in vivo disease model and it clearly delayed tumor growth in TNBC xenografts implanted nude mice^18^. Given the importance of SMYD2 in TNBC development, SMYD2 should become an attractive candidate for the development of targeted therapy specific for TNBC.

## Materials and methods

### Cell culture and reagents

Human breast cancer cell lines MCF-7, MDA-MB231, MDA-MB468, Hs578T and non-tumorigenic MCF10A human mammary epithelial cells as well as Human Embryonic Kidney 293 cells (HEK293) were obtained from American Type Culture Collection. T47D cells was kindly provided by Dr. Joan Lewis-Wambi and Dr. Christy Hagan at the University of Kansas Medical Center (KUMC) (Kansas City, Kansas, USA). All of the cells were maintained according to the recommended protocol. For knockdown of SMYD2, cell lines were infected with lentiviral pGIPZ shRNA vector containing short hairpins and GFP reporter (Open Biosystems, Pittsburgh, PA, USA). AZ505 was purchased from MedChem Express and dissolved in DMSO (Sigma) at a stock solution of 20 mM. S3I-201 was purchased from Sigma and dissolved in DMSO (Sigma) at a stock solution of 100 mM. BAY-11-7085 was purchased from Cayman Chemical and dissolved in DMSO (Sigma) at a stock solution of 40 mM. All the stock solutions were stored at −20 °C. TNF-α and IL-6 were purchased from Sigma-Aldrich. pAcGFP1-C1-SMYD2 and pAcGFP1-C1 plasmids were purchased from Clonetech.

### Western blot and immunoprecipitation

We performed immunoprecipitation and Western blotting on whole-cell lysates as previous described^49^. Briefly, cell pellets were collected and re-suspended in lysis buffer (20 mM Tris-HCl, pH 7.4, 150 mM NaCl, 10% glycerol, 1% Triton X-100, 1 mM Na_3_VO_4_, 25 mM β-glycerol-phosphate, 0.1 mM PMSF, Roche complete protease inhibitor set and Sigma phosphatase inhibitor set). The re-suspended cell pellet was vortexed for 20 s and then incubated on ice for 30 min and centrifuged at 20,000*g* for 30 min. The protein in the cell lysates were subjected to Western blot analysis or immunoprecipitation.

For SMYD2 immunoprecipitation, anti-SMYD2 antibody or control IgG was coupled to protein A agarose bead (Pierce) in PBS containing 5 mg/ml bovine serum albumin (Sigma) for 6 h at 4 °C on a rotating platform. The cell lysates were then incubated with the beads coupled with SMYD2 antibody or control IgG overnight at 4 °C. The next day, beads were washed with lysis buffer containing additional 300 mM NaCl and the immunoprecipitants were eluted off the beads using loading buffer with boiling for 5 min.

The antibodies used for Western analysis included: anti-SMYD2, anti-STAT3, anti-p65 antibodies (Santa Cruz,1:500 dilution), anti-actin antibody (Sigma, 1:5000 dilution), anti-phospho-STAT3, anti-phospho-p65, anti-phospho-S6, anti-phospho-AKT, anti-phospho-ERK, anti-S6, anti-AKT, and anti-ERK antibodies (Cell Signaling Technologies; 1:1000 dilution). All primary antibodies were used at 1:50 dilution for immunoprecipitation and for Western blotting as indicated above. Donkey-anti-rabbit IgG-horseradish peroxidase, Donkey-anti-goat IgG-horseradish peroxidase and Goat anti-mouse IgG-horseradish peroxidase (Santa Cruz, 1:8000 dilution) were used as secondary antibodies.

### Immunohistochemistry

Xenograft tissues were fixed with 4% paraformaldehyde (pH 7.4). For Ki67 staining, a monoclonal mouse anti-Ki67 antibody (Cell Signaling Technologies; 1:1000 dilution), a biotinylated secondary antibody (Sigma-Aldrich; 1:100 dilution), and DAB substrate system were used. Then sections were counter stained by hematoxylin. Images were analyzed with a NIKON ECLIPSE 80i microscope.

### Immunofluorescence staining

Breast cancer cell lines as well as MCF10A cells were detected by immunofluorescence staining (IF staining) with anti-SMYD2 antibody. Cultured cells were grown on sterile glass cover slips overnight at 37° C. The cells were washed briefly with PBS and then fixed for 10 min in −20 °C methanol, 2 min in cold acetone. After that the cells were washed with PBS for three times. Use suction to remove reagents after each step, but avoid drying of specimens between steps. Incubate with primary antibody for 60 min. The antibody is used at 1 μg/ml with 2% BSA in PBS. Wash three times with PBS for 5 min each. Incubate with secondary antibody, Fluro-488 anti-goat IgG, for 60 min Wash three times with PBS for 5 min each and then the cover slips were mounted in Prolong Gold Anti-fade reagent with DAPI (Invitrogen). Images were analyzed using a NIKON ECLIPSE 80i microscope.

### SMYD2 knockdown by lentivirus carrying SMYD2 shRNA

HEK293T cells were transfected either with lentiviral plasmid pGIPZ-SMYD2 (Open Biosystems), carrying *SMYD2* shRNA, or with control empty vector pGIPZ-NS, plus psPAX2 packaging plasmid and pMD2.G envelope plasmid using calcium phosphate. After transfection for 16 h, the medium containing the transfection reagent was removed and replaced with fresh complete DMEM plus 10% FBS and penicillin/streptomycin. The lentiviral particles were harvested from HEK293T cells after another 48 h. MDA-MB231 and MDA-MB468 cells were then infected with appropriate amounts of lentiviral particles together with 5 μg/ml polybrene (Sigma-Aldrich) for 24 h, and then virus-containing medium was removed and replaced with fresh medium plus 10 μg/ml puromycin. After 48 h of puromycin selection, all remaining cells were GFP positive, as detected by microscopy. MDA-MB231 and MDA-MB468 cells were harvested after lentiviral particle infection for 5 days and analyzed by RT-PCR to examine the efficiency of SMYD2 knockdown. STAT3 knockdown was performed in the same way.

### RNA interference

The RNA oligonucleotides that specifically targeted human SMYD2 was purchased from Santa Cruz Biotechnology, Inc. The RNA oligonucleotides were transfected with DharmaFECT siRNA transfection reagent (Dharmacon). 24 and 48 h after transfection, cells were harvested and analyzed by Western blotting.

### Quantitative reverse-transcription polymerase chain reaction (qRT-PCR)

Total RNA was extracted using the RNeasy plus mini kit (Qiagen). Total RNA (1 μg) was used for RT reactions in a 20-μl reaction to synthesize cDNA using Iscript cDNA Synthesis Kit (BioRad). RNA expression profiles were analyzed by real-time PCR using iTaq SYBER Green Supermix with ROX (BioRad) in an icycleriQTM Real-time PCR detection system. The complete reactions were subjected to the following program of thermal cycling: 40 cycles of 10 s at 95 °C and 20 s at 60 °C. A melting curve was run after the PCR cycles, followed by a cooling step. Each sample was run in triplicate in each experiment, and each experiment was repeated 3 times. Expression levels target genes were normalized to the expression level of GAPDH. All the primers used were listed in Table S1.

### Chromatin immunoprecipitation (CHIP) assay

ChIP assay was performed according to the protocol^50^. Chromatin DNA was subjected to IP with anti-Smyd2, anti-STAT3, anti-p65 antibodies and anti–H3K4-me2 antibody (ab7766; Abcam), or normal rabbit IgG and then washed, after which the DNA-protein cross-links were reversed. The recovered DNA was analyzed by PCR for the binding of STAT3, p65, and H3K4-me at the mouse Smyd2 promoter.

### Terminal deoxynucleotidyl transferase-mediated dUTP nick end-labeling (TUNEL) assay

TUNEL assays for AZ505-treated or SMYD2 knockdown cells and for AZ505–treated or shRNA-SMYD2 knockdown xenografts were performed according to the manufacturer’s protocols (In Situ Death Detection Kit; Roche). Prolong Gold Anti-fade reagent with DAPI (Invitrogen) was used. Immunofluorescence images were obtained with a NIKON ECLIPSE 80i microscope.

### Cell cycle analysis

A total of 150,000 cells were seeded in six-well plates and allowed to recover for 16-24 h, cells were then starved with serum-free medium for 24 h. Then cells were left untreated or treated with AZ505 for 24 h. The cells were pelleted and washed by adding 1 ml PBS; the cells were centrifuged at 2000 rpm for 5 min and the supernatant was aspirated; 1 ml of cold 70% ethanol was added drop by drop into the cell pellet to re-suspend the cells. The cells were then incubated at −20 °C overnight. After that the cells were washed twice in 1 × PBS to remove the ethanol, then centrifuged for 5 min at 3000 rpm and aspirate the supernatant. For PI staining, the cells were suspended in 0.5 ml of PI buffer (containing RNase and Triton X-100) for 10–15 min at room temperature. The cells were kept away from light at 4 °C prior to further analysis on the flow cytometer which took place within 1 h. FACS data were analyzed with FlowJo software.

### Transfection assays

HEK293 T cells were cultured to confluence in DMEM media containing 10% fetal calf serum (FetalcloneIII, Clonetech). The control or SMYD2 overexpression pAcGFPC1 vectors were transfected into cells using the Lipofectamine 2000 Kit according to the manufacturer’s recommendations. Transfected cells were maintained at 37 °C/5%CO2 for 24 or 48 h.

### MTT assays

Cell proliferation was measured using MTT-based kit (Promega), according to the manufacturer’s instructions.

### Wound closure assay

MDA-MB231 cells or MDA-MB468 cells (4 × 10^5^ cells/2 ml) were seeded in a 6-well plate and incubated at 37 °C until 90 to 100% confluent. Wounds were generated on the monolayer of cells with sterile 10 μl pipet tip and washed with PBS to take images at T_0,_ then cells were switched to the serum containing normal medium with different concentrations of AZ505 for 24 h. Pictures of same position of each well were taken at 24 h (T_24_). We performed the wound closure assay with MCF-7 cells and T47D cells in a similar way, but pictures were taken at zero (T_0_) and 48 h (T_48_), due to the cell growth rates.

### In vitro methylation assay

We made constructs for GST-SMYD2, GST-STAT3 and GST-p65 as well as the lysine site mutants of STAT3 and p65, induced expression in the presence of IPTG, and purified the proteins by glutathione agarose beads. In vitro methylation assays were performed using 1–2 μg of recombinant proteins incubated with 1 μg of recombinant GST-SMYD2 and 2 μCi ^3^H-AdoMet (Perkin Elmer) in buffer containing 50 mM Tris-HCl (pH 8.0), 10% glycerol, 20 mM KCl, 5 mM MgCl2, and 1 mM PMSF at room temperature overnight. The reaction mixture was resolved by SDS–PAGE followed by autoradiography.

### Tumorigenesis and treatment assay

All animal works were done in accordance with approved protocol by the Institutional Animal Care and Use Committee (IACUC) of the University of Kansas Medical Center. Animals were maintained and treated under pathogen-free conditions. Female NU/NU nude (Charles River) of 4–6 weeks old were used in xenograft studies orthotopic mammary fat pad tumor assays. One shRNA construct showed most effective knockdown of SMYD2 and subsequently used to knockdown SMYD2 in TNBC MDA-MB231 cells as well as the control shRNA cells that were used for animal studies. For orthotopic tumor assays, cells were collected in PBS and inoculated into the right mammary fat pad (5 × 10^7^/ml, 0.1 ml per mouse, *n* = 6 in each group). The primary tumor outgrowth was monitored every two or three days with caliper measurements. Tumor volumes were calculated according to the following equation: Volume = (width)^2^ × length/2. The animals in both groups were euthanized when the tumors reached an average length of 1 cm in control group, and tumors were removed and weighed.

For treatment assay, all the mice were inoculated with MDA-MB231cells (5 × 10^7^/ml, 0.1 ml per mouse, *n* = 10 in each group) or MDA-MB468 cells (5 × 10^7^/ml, 0.1 ml per mouse, *n* = 5 in each group) via mammary fat pad, tumor growth was monitored. When tumors reached an average volume of about 100 mm^3^ (50~200 mm^3^) in size, then mice were randomized into two groups, DMSO control and SMYD2 inhibitor AZ505 treatment group. AZ505 and DMSO control were administered intraperitoneally at 40 mg/ kg/d. Tumor size and body weight were recorded every 2 days. Animals were euthanized 2 weeks after the first treatment and tumors were removed and weighed.

### TCGA data analysis

All TCGA data and figures were accessed, analyzed and generated using the cBio Cancer Genomics Portal. All data included in this manuscript is in agreement with the TCGA publication guidelines.

### Statistics

All data are presented as mean ± SEM. All statistical analyses were performed using SPSS Statistics 22 software. P-values were calculated by two-tailed unpaired Student’s *t*-test, one-way ANOVA and *p < *0.05 was considered significant.

### Study approval

All animal protocols were approved and conducted in accordance with Laboratory Animal Resources of KUMC (Kansas City, Kansas, USA) and Institutional Animal Care and Use Committee regulations (Protocol # 2015-2290).

## Electronic supplementary material

Supplementary Figure legends and table

Suppelemntary Figure S1

Supplementary Figure S2

Supplementary Figure S3

Supplementary Figure S4

Supplementary Figure S5
