# Supplementary material for: Lysine methyltransferase SMYD2 promotes triple negative breast cancer progression
Source: Cell Death Dis. 2018 Feb 27;9(3):326. doi: 10.1038/s41419-018-0347-x (PMC5832424; doi:10.1038/s41419-018-0347-x)
Supplement: Supplementary file 1 — Supplementary Figure legends and table [file 41419_2018_347_MOESM1_ESM.doc]

**Supplemental data**

**Supplementary Figure Legends**

**Supplementary Figure 1. SMYD2 contributes to cell proliferation. (A** and **B)** Overexpression of GFP-tagged SMYD2 increased the proliferation of both HEK293T cells (**A**) and MCF10A cells (**B**) as examined by MTT assay. (**C**) The specificity of AZ505 was tested in the primary renal epithelial cells isolated from the kidneys of Smyd2 wild type and kidney-specific knockout mice. Western blotting showed the protein levels in these mice. Representative result from at least three independent experiments was shown.

**Supplementary Figure 2. Inhibition of SMYD2 with AZ505 suppressed ER positive breast cancer cells proliferation and migration.** (**A**) Treatment with AZ505 (24 hours) inhibited cell proliferation of MCF-7 cells (*top panel*) and T47D cells (*bottom panel*) in a dose dependent manner as examined by MTT assay. (**B**) FACS analysis indicated that inhibition of SMYD2 with AZ505 decreased S phase entry in MCF-7 cells (*left panel*) and T47D cells (*right panel*). (**C**) Wound closure assay indicated that AZ505 treatment (20μM, 48 hours) suppressed cell migration of MCF-7 cells (*left panel*) and T47D cells (*right panel*).

**Supplementary Figure 3. SMYD2 regulates the methylation and activation of STAT3 and p65 in MDA-MB468 cells.** (**A**) Knockdown of SMYD2 with shRNA decreased the methylation and phosphorylation of STAT3 and p65, but did not affect their expression in MDA-MB468 cells. Representative result from at least two independent experiments was shown. (**B**) Overexpression of GFP-tagged SMYD2 increased the methylation and phosphorylation of STAT3 and p65 but did not affect the expression of STAT3 and p65 in HEK293T cells. (**C**) Inhibition of SMYD2 with AZ505 decreased the methylation and phosphorylation of STAT3 and p65, but did not affect their expression in MDA-MB468 cells. Representative result from at least two independent experiments was shown.

**Supplementary Figure 4. SMYD2 methylates STAT3 and P65 in vitro.** (**A**) In vitro methylation assay on full-length recombinant STAT3, STAT3-K49R, K140R and K685R with recombinant SMYD2. Autoradiograms of the methylation assay indicated that SMYD2 methylated the recombinant full-length STAT3, STAT3-K49R and K140R but not K685R after long exposure (*left panel*). Coomassie stain indicated the expression of recombinant proteins (*right panel*). (**B**) In vitro methylation assay on full-length recombinant p65 (RelA), p65-K218R, K221R and K310R with recombinant SMYD2. Autoradiograms of the methylation assay indicated that SMYD2 methylated the recombinant full-length p65 (RelA), p65-K218R and K221R but not K310R (*left panel*). Coomassie stain indicated the expression of recombinant proteins (*right panel*). Histone H3 was used as a positive control in all the in vitro methylation assays.

**Supplementary Figure 5. PTPN13 is downregulated by SMYD2 in TNBCs.** (**A**) qRT-PCR analysis of mRNA expression of PTPN13 in MCF10A cells compared to that in MDA-MB231 cells and MDA-MB468 cells. (**B**) qRT-PCR analysis of mRNA expression of PTPN13 in MDA-MB468 cells treated with SMYD2 shRNA or AZ505 compared to the controls. (**C**) Western blot analysis of PTPN13 in SMYD2 knockdown or AZ505 treated MDA-MB468 cells. (**D** and **E**) Western blot analysis of the phosphorylation of ERK, AKT, S6, and RB as well as the phosphorylation of STAT3 and p65 in MDA-MB231 cells or MDA-MB468 cells treated with or without AZ505.

**Supplementary Table 1. The primers used for quantitative real time PCR and ChIP-PCR.**

| **Gene name** | **Forward (5’- 3’)** | **Reverse (5’- 3’)** |
| --- | --- | --- |
| *SMYD2* | TGTGTTTGAGGACAGTAACGTG | GAGGGAGTACAAAGGATAGTGC |
| *SMYD2-Promoter* | AACCCTCTGCACACCAAACTTC | GGAACGCCAAGGAGAAAGC |
| *GAPDH* | GCACCGTCAAGGCTGAGAAC | AGGGATCTCGCTCCTGGAA |
| *IL32* | CCTTGGCTCCTTGAACTTTTG | CTGTCCACGTCCTGATTCTG |
| *SOX2* | CACACTGCCCCTCTCAC | TCCATGCTGTTTCTTACTCTCC |
| *BATF* | GACAGAGGCAGACACAGAAG | TGCTTGATCTCCTTGCGTAG |
| *FOXO1* | AGACAACGACACATAGCTGG | AGGGAGTTGGTGAAAGACATC |
| *CDC25A* | TGTTGAAGAGACCAGAACGATC | GGGAAGATGCCAGGGATAAAG |
| *FAS* | AAGCTCTTTCACTTCGGAGG | GGGCATTAACACTTTTGGACG |
| *BCL2* | GTGGATGACTGAGTACCTGAAC | GCCAGGAGAAATCAAACAGAGG |
| *TNFAIP3* | GATAGAAATCCCCGTCCAAGG | CTGCCATTTCTTGTACTCATGC |
| *IL6* | CCACTCACCTCTTCAGAACG | CATCTTTGGAAGGTTCAGGTTG |
| *Cyclin D1* | CATCTACACCGACAACTCCATC | TCTGGCATTTTGGAGAGGAAG |
| *BCL2L1* | GTGGAAAGCGTAGACAAGGAG | CTGCATTGTTCCCATAGAGTTC |
| *IL2* | AAAGAAAACACAGCTACAACTGG | GAAGATGTTTCAGTTCTGTGGC |
| *TNF* | ACTTTGGAGTGATCGGCC | GCTTGAGGGTTTGCTACAAC |
