# Supplementary figures and images for: Lysine methyltransferase SMYD2 promotes triple negative breast cancer progression
Source: Cell Death Dis. 2018 Feb 27;9(3):326. doi: 10.1038/s41419-018-0347-x (PMC5832424; doi:10.1038/s41419-018-0347-x)

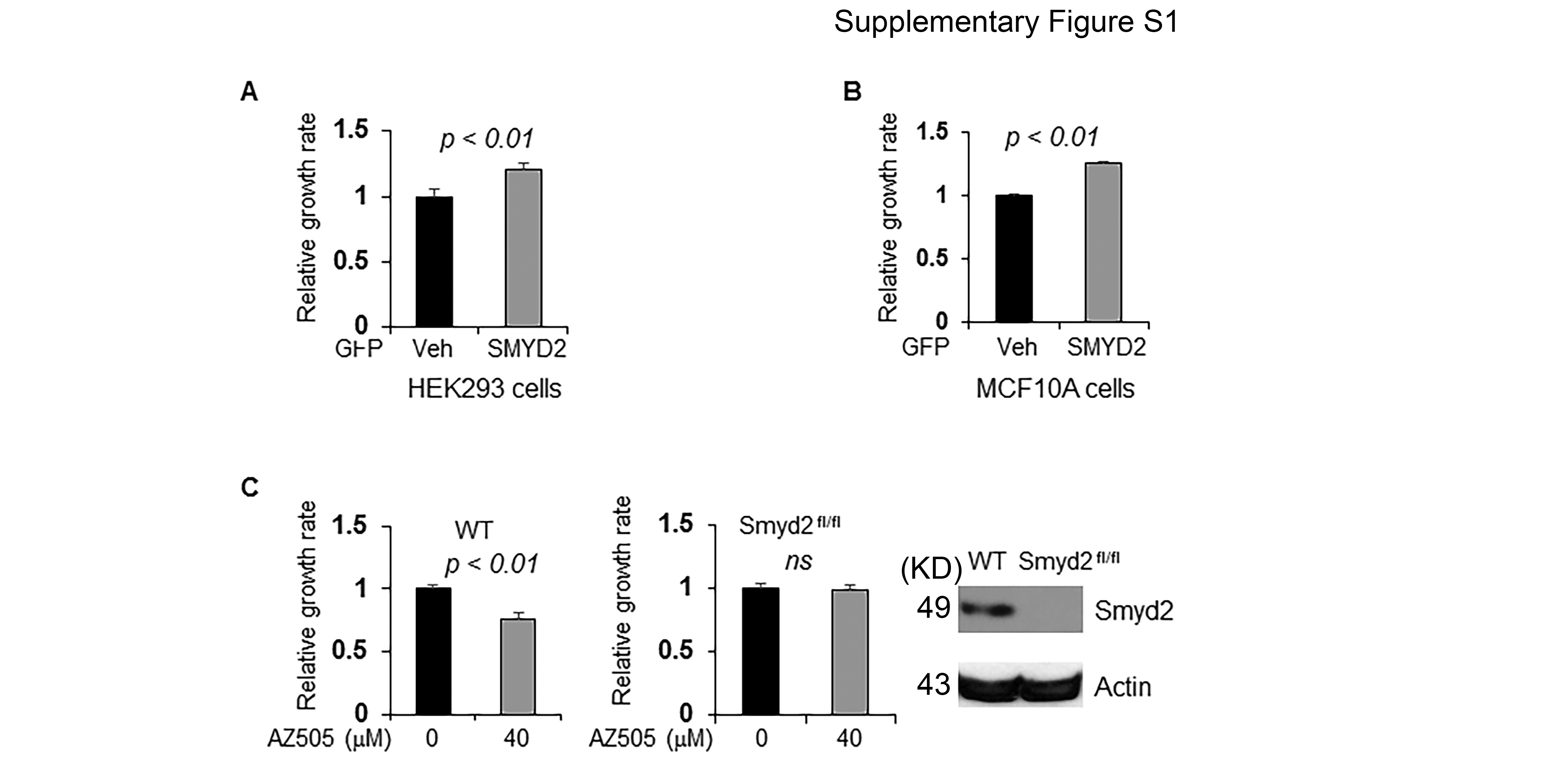

Supplement: Supplementary file 2 — Suppelemntary Figure S1 [file 41419_2018_347_MOESM2_ESM.jpg]

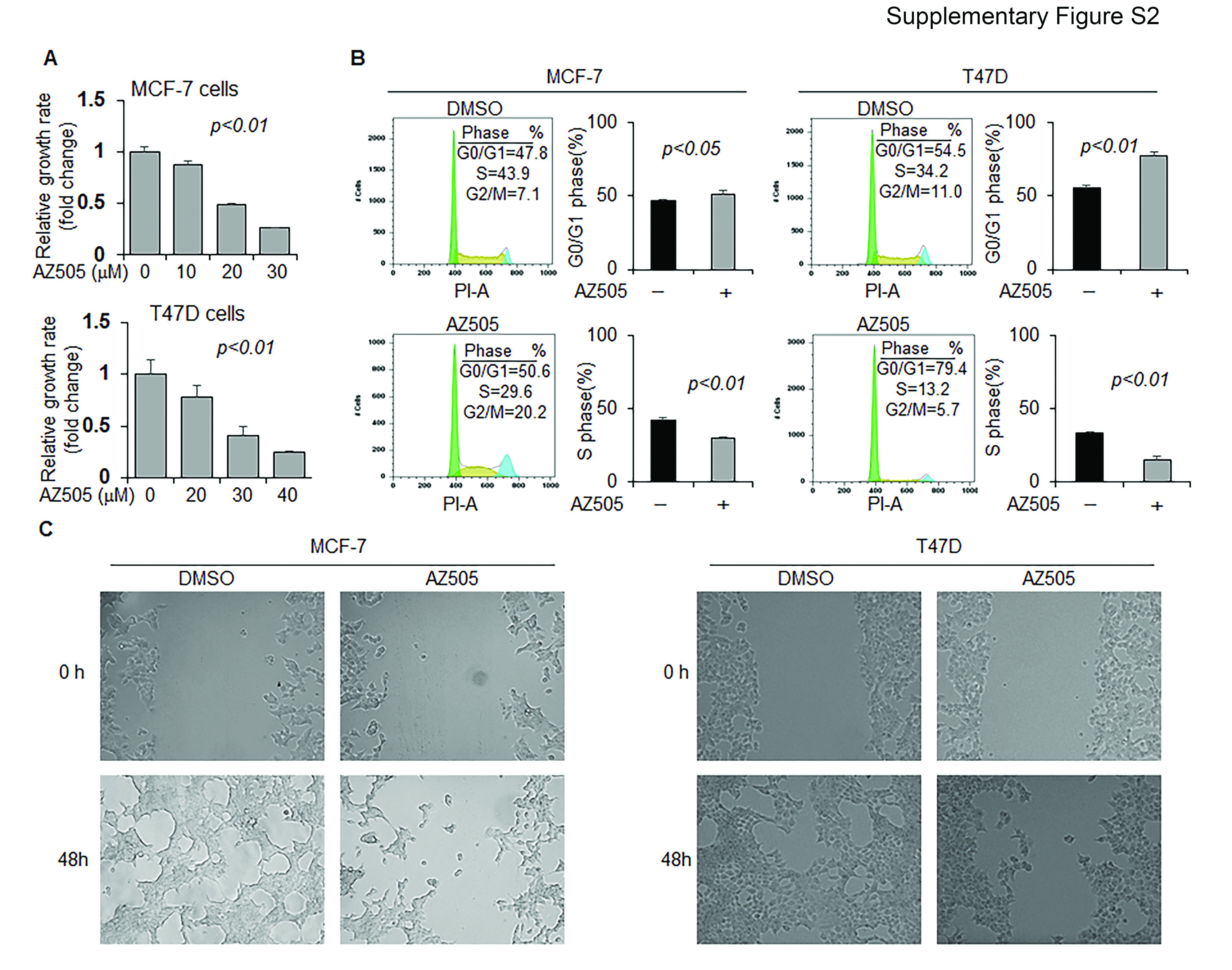

Supplement: Supplementary file 3 — Supplementary Figure S2 [file 41419_2018_347_MOESM3_ESM.jpg]

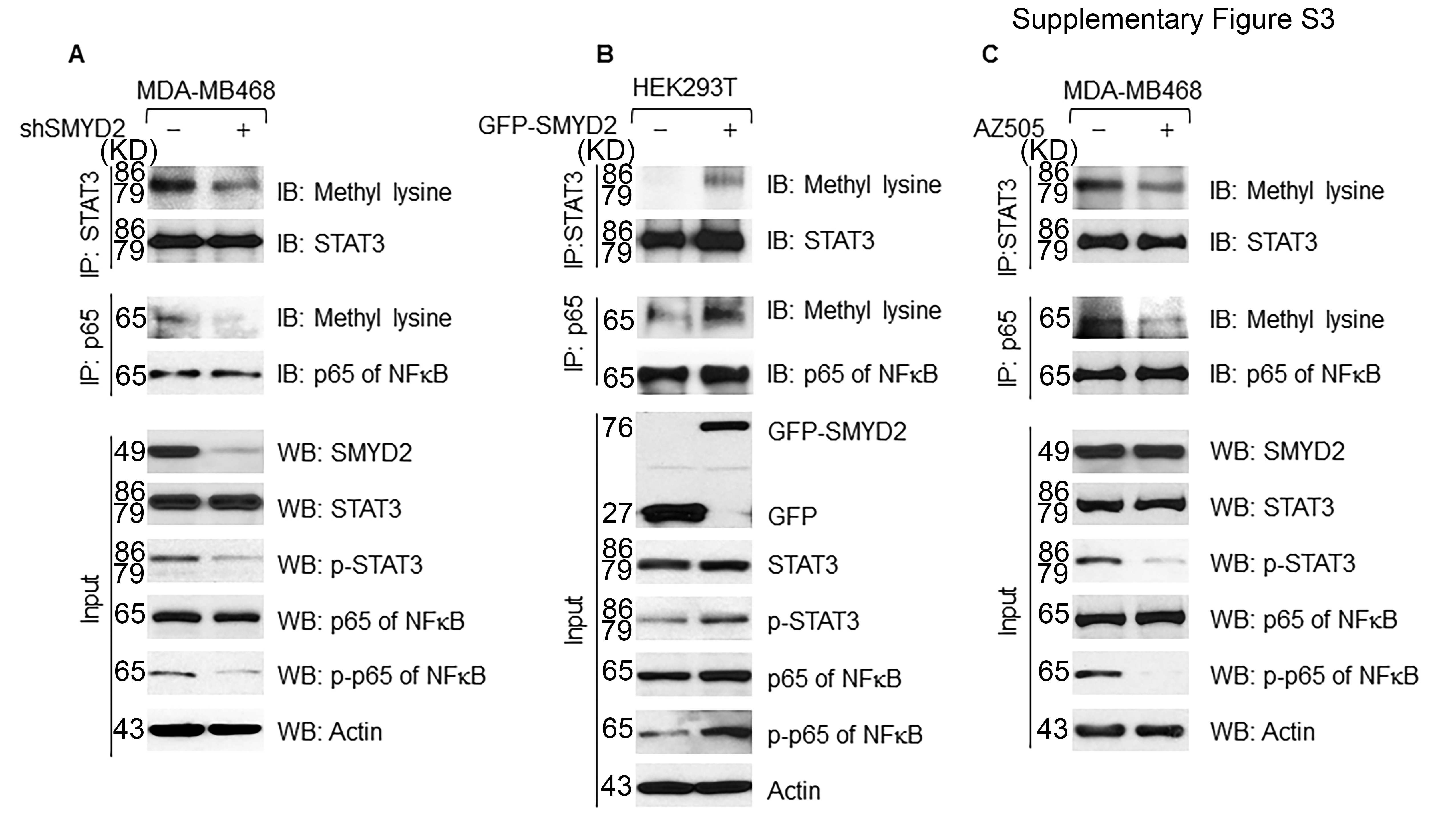

Supplement: Supplementary file 4 — Supplementary Figure S3 [file 41419_2018_347_MOESM4_ESM.jpg]

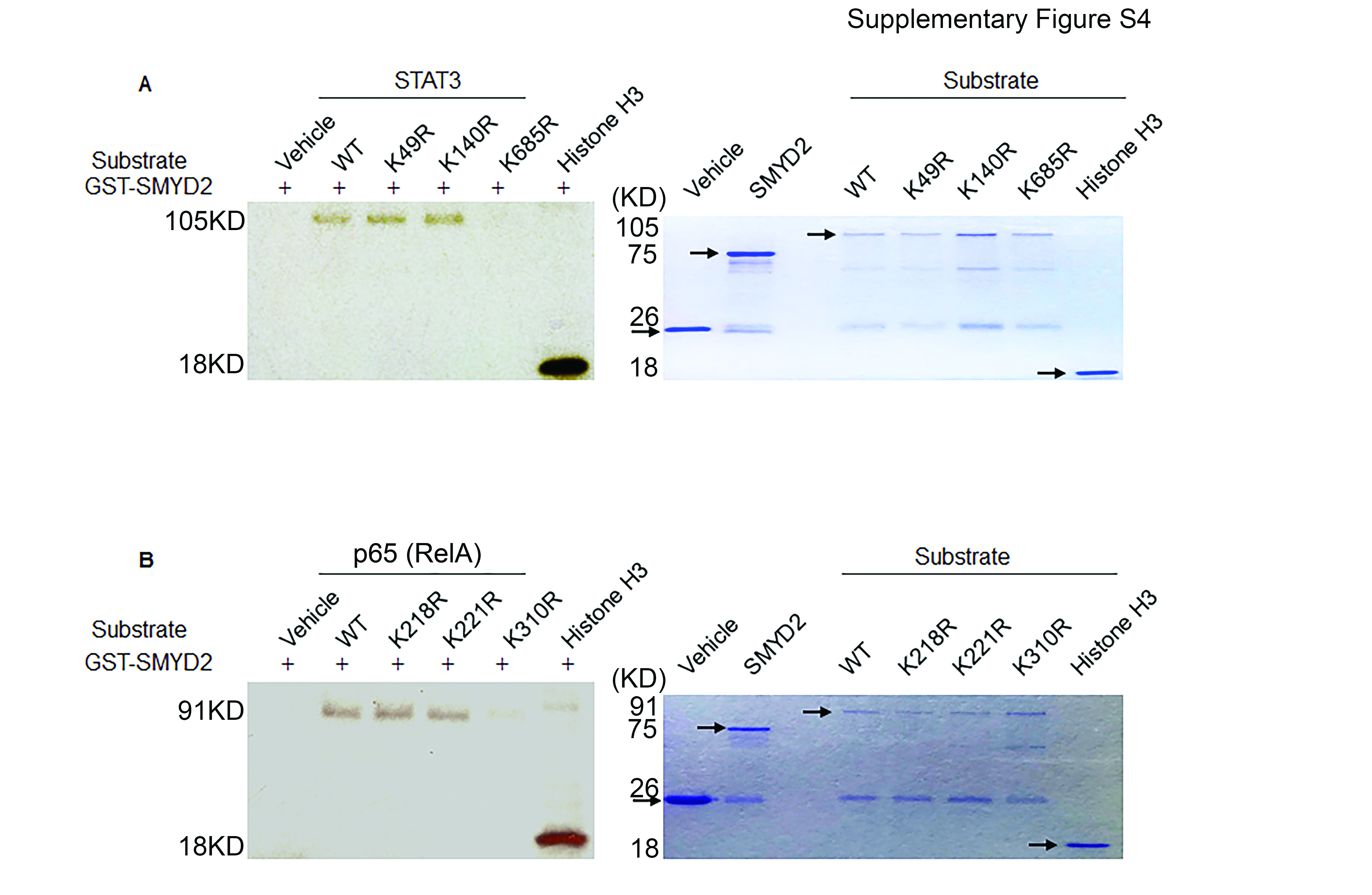

Supplement: Supplementary file 5 — Supplementary Figure S4 [file 41419_2018_347_MOESM5_ESM.jpg]

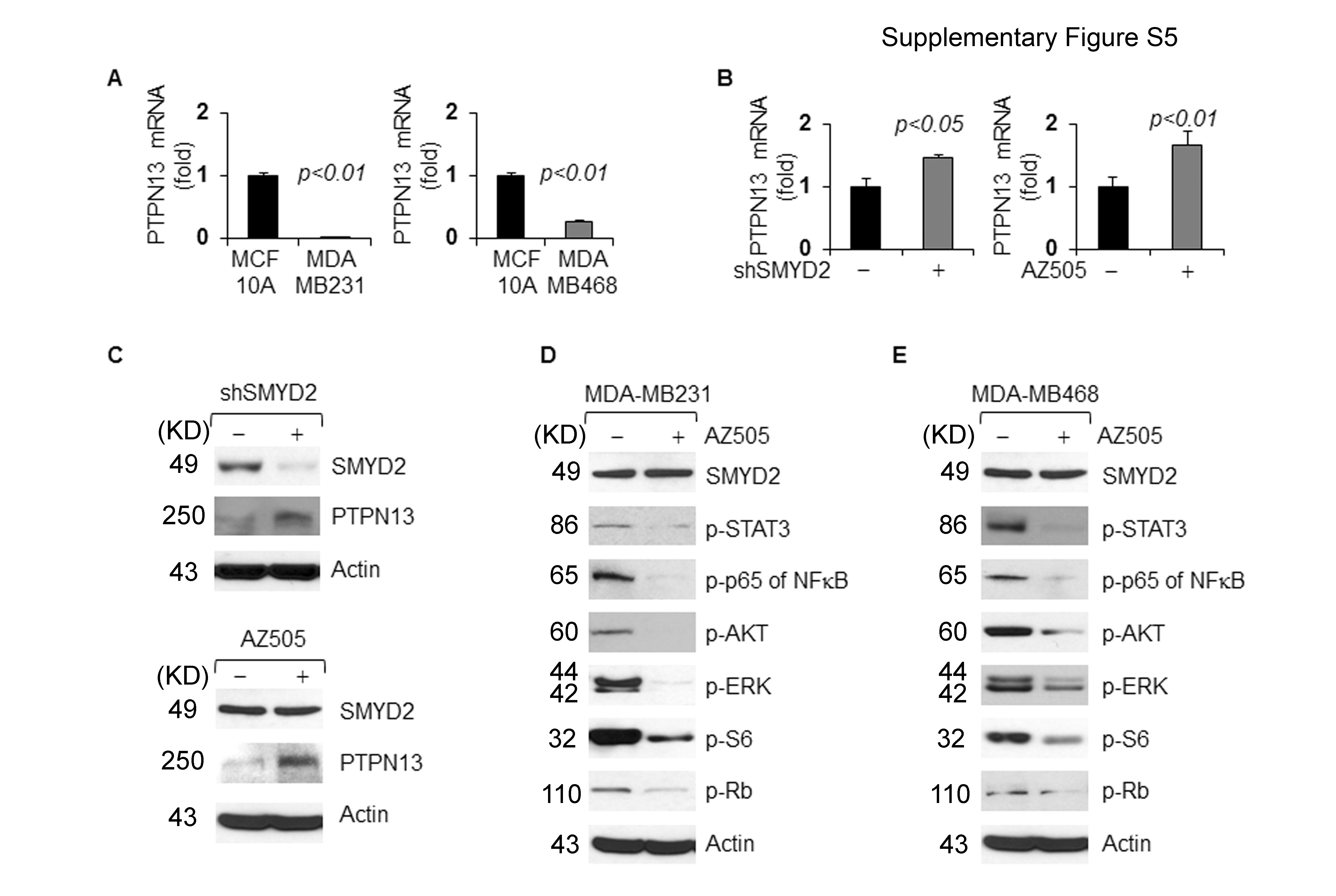

Supplement: Supplementary file 6 — Supplementary Figure S5 [file 41419_2018_347_MOESM6_ESM.jpg]
